# Nanozyme‐Integrated Hydrogel Targeting AGEs for Diabetic Osteoarthritis Therapy

**DOI:** 10.1002/advs.202516389

**Published:** 2025-12-05

**Authors:** Rui Chen, Yanguo Su, Guiyuan Zhao, Jiaojiao Tao, Qijie Diao, Guangli Xiang, Tianze Jiang, Lu Han, Xia Zhao

**Affiliations:** ^1^ Key Laboratory of Marine Drugs Ministry of Education Shandong Key Laboratory of Glycoscience and Glycotherapeutics School of Medicine and Pharmacy Ocean University of China Qingdao 266003 China; ^2^ Laboratory for Marine Drugs and Bioproducts Qingdao Marine Science and Technology Center Qingdao 266237 China

**Keywords:** cartilage repair, heat shock protein 70, mild photothermal therapy, nanozymes, synovitis

## Abstract

Advanced glycation end‐products (AGEs) play a crucial role in the pathogenesis of diabetic osteoarthritis (DOA), contributing to cartilage degradation and impaired joint lubrication, which complicate clinical management. However, no therapeutics specifically targeting AGEs are developed for DOA treatment. Here, a composite hydrogel (PTC‐MP) incorporated with polydopamine‐coated tannic cerium (PTC) nanozymes and magnesium ions (Mg^2+^), implementing a strategy of “restrain‐restore‐reinforce (3R)” for AGEs‐directed DOA therapy is reported. PTC‐MP hydrogel effectively restrains the formation of AGEs by scavenging free radicals, chelating ferrous ions, and competitive hydrophobic site binding. By leveraging mild photothermal therapy (mPTT), PTC‐MP hydrogel restores cartilage homeostasis by reducing AGEs‐induced reactive oxygen species (ROS) overproduction and mitochondrial dysfunction. Furthermore, Mg^2+^ in PTC‐MP hydrogel reinforces joint repair by stimulating endogenous hyaluronic acid (HA) secretion to improve lubrication. In a rat DOA model, PTC‐MP hydrogel with mPTT significantly attenuated DOA progression, reduced osteophytes formation and synovial inflammation, and improved motor function. Therefore, the “3R” strategy targeting AGEs provides a promising therapeutic approach for recalcitrant DOA.

## Graphical abstract

A nanozyme–integrated hydrogel (PTC‐MP) exhibiting with mild photothermal effects is constructed, and a “3R” (restrain‐restore‐reinforce) strategy is proposed for diabetic osteoarthritis (DOA) treatment based on advanced glycation end products (AGEs) targeting. PTC‐MP hydrogel effectively restrains the formation of AGEs, restores cartilage homeostasis and reinforces DOA repair.

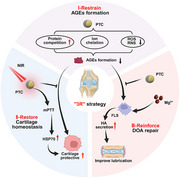

## 1 Introduction

Osteoarthritis (OA) is a common degenerative joint disease that seriously affecting the life quality of patients.^[^ ^1^ ^]^ Recent studies have found that diabetes is an independent risk factor for OA. Especially in elderly diabetic patients, OA is a common concomitant disease, which is called diabetic osteoarthritis (DOA).^[^ ^2^ ^]^ The special chronic inflammatory microenvironment in diabetic patients makes the treatment of DOA difficult,^[^ ^3^ ^]^ which can cause pain and functional disorders, affect mental health, increase financial burden and shorten life expectancy.^[^ ^4^ ^]^

AGEs are complex compounds generated by a series of non‐enzymatic reactions between reducing sugars and proteins,^[^ ^5^ ^]^ which play a key role in the pathogenesis of DOA.^[^ ^6^ ^]^ Clinical studies have shown that the level of AGEs in human articular cartilage is significantly higher than that in other tissues,^[^ ^7^ ^]^ and is highly correlated with the severity of OA.^[^ ^8^ ^]^ In a clinical survey of 84 patients with knee OA (including 46 diabetic patients and 38 non‐diabetic patients), the accumulation of AGEs driven by hyperglycemia significantly accelerated the progression of OA in diabetic patients.^[^ ^9^ ^]^ In addition, the accumulation of AGEs was observed in the synovial tissue of both diabetic patients and diabetic model animals, leading to the aggravation of arthritis.^[^ ^10^, ^11^ ^]^ Additionally, AGEs are prone to accumulate in joints and cause oxidative stress and mitochondrial dysfunction of chondrocytes,^[^ ^12^ ^]^ which lead to cartilage destruction. Moreover, AGEs can stimulate fibroblast‐like synovial cells (FLS) to release inflammatory factors, such as tumor necrosis factor‐α (TNF‐α), interleukin‐6 (IL‐6) and matrix metalloproteinase‐13 (MMP‐13), which lead to lubrication dysfunction and exacerbate the progression of OA.^[^ ^13^ ^]^ Therefore, the accumulation of AGEs, cartilage destruction, and lubrication dysfunction are important reasons for the difficult treatment of DOA. However, the current clinical interventions are still the use of analgesics or intra‐articular injection of steroids,^[^ ^14^ ^]^ which can alleviate symptoms but cannot effectively solve the chronic inflammation caused by AGEs in the joints. Therefore, there is an urgent need to develop new strategies targeting AGEs to treat DOA.

Reducing AGEs is considered as an effective strategy to control diabetic complications.^[^ ^15^ ^]^ Aminoguanidine (AG) was once considered to be an effective anti‐AGES drug in clinical practice,^[^ ^16^ ^]^ but its high toxicity was observed during long‐term administration, and its clinical trial was forced to be terminated.^[^ ^17^ ^]^ Currently, there are almost no anti‐AGES drugs have been used clinically for DOA treatment. Recent studies have found that tannic acid (TA) can reduce the formation of AGEs,^[^ ^18^ ^]^ showing the therapeutic potential against diabetic complications. However, the accumulated AGEs can still increase the level of reactive oxygen species (ROS) in chondrocytes and induce cell death,^[^ ^12^ ^]^ which is difficult to effectively alleviate inflammation.

Cerium (Ce) ‐based nanozymes can clear ROS and restore mitochondrial function by reversible conversion between Ce^4+^ (oxidation)/Ce^3+^ (reduction), which has shown potential for OA treatment in recent years.^[^ ^19^, ^20^, ^21^, ^22^ ^]^ However, most nanozymes have the problem of insufficient cellular uptake and enzyme activity. Studies have shown that mild photothermal therapy (mPTT) can effectively improve the cellular uptake and enzyme activity of nanozymes.^[^ ^23^, ^24^ ^]^ In particular, the heat shock protein 70 (HSP 70) induced by mPTT plays an important role in the treatment of diabetic complications and OA.^[^ ^25^, ^26^ ^]^ It has been reported that polydopamine (PDA) modified materials can achieve mPTT under near‐infrared (NIR, 808 nm) irradiation.^[^ ^27^, ^28^ ^]^ Therefore, the combination of PDA‐modified cerium nanozymes with mPTT will be beneficial for the treatment of DOA.

Insufficient joint lubrication caused by AGEs is another major factor that exacerbates DOA. Hyaluronic acid (HA) secreted by fibroblast synovial cells (FLS) can improve joint lubrication and maintain the homeostasis of joint cavity. However, AGEs can induce cellular ROS to inhibit the activity of hyaluronic acid synthase 2 (HAS‐2), thereby reducing endogenous HA secretion and exacerbating joint wear.^[^ ^29^ ^]^ It has been reported that magnesium ion (Mg^2+^) can effectively catalyze the synthesis of intracellular HA,^[^ ^30^ ^]^ and cerium‐based nanozymes can remove ROS and protect FLS to provide a favorable microenvironment for HA synthesis.^[^ ^20^ ^]^

**Scheme 1:**
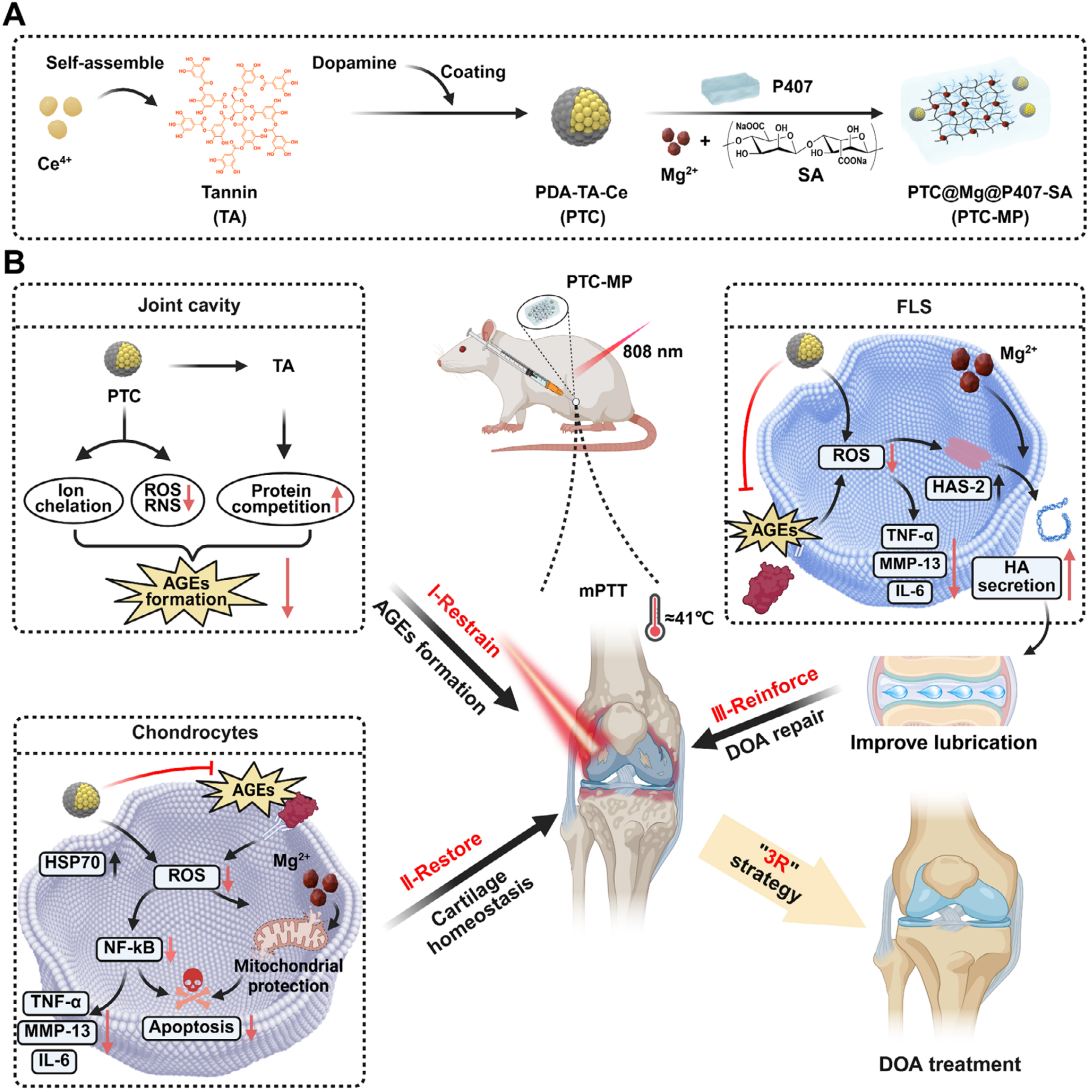
Design and mechanism of PTC‐MP hydrogel for AGEs‐directed DOA therapy. A) Schematic illustration for PTC‐MP hydrogel preparation. B) PTC‐MP hydrogel with mPTT for DOA treatment targeting AGEs and "3R" strategy. I‐PTC‐MP hydrogel restrains the formation of AGEs by scavenging free radicals, chelating ferrous ions, and competitive hydrophobic site binding; II‐By leveraging mPTT, PTC‐MP hydrogel restores cartilage homeostasis by reducing AGEs‐induced ROS overproduction and mitochondrial dysfunction; III‐PTC‐MP hydrogel reinforces DOA repair by promoting endogenous HA secretion and improving joint lubrication (Created with BioRender.com).

Here, we develop a nanozyme–integrated hydrogel (PTC‐MP) exhibiting mPTT effects for the treatment of AGEs‐directed DOA. The hydrogel was constructed by incorporating PDA‐modified TA‐cerium nanozymes (PTC) into the interpenetrating network system formed by sodium alginate (SA) chelated with Mg^2+^ ions and Poloxam 407 (**Scheme** **1**A). We hypothesize that TA in PTC‐MP hydrogel can restrain AGEs formation, PTC can cooperate with mPTT to restore cartilage homeostasis by reducing AGEs‐induced ROS overproduction, and Mg^2+^ can reinforce DOA repair by promoting endogenous HA secretion to improve joint lubrication (Scheme 1B). Therefore, we anticipate that PTC‐MP hydrogel will significantly mitigate DOA progression by simultaneously targeting AGEs accumulation and its downstream pathological consequences.

## 2 Results and Discussion

### 2.1 Synthesis and Characterization of PTC

**Figure 1:**
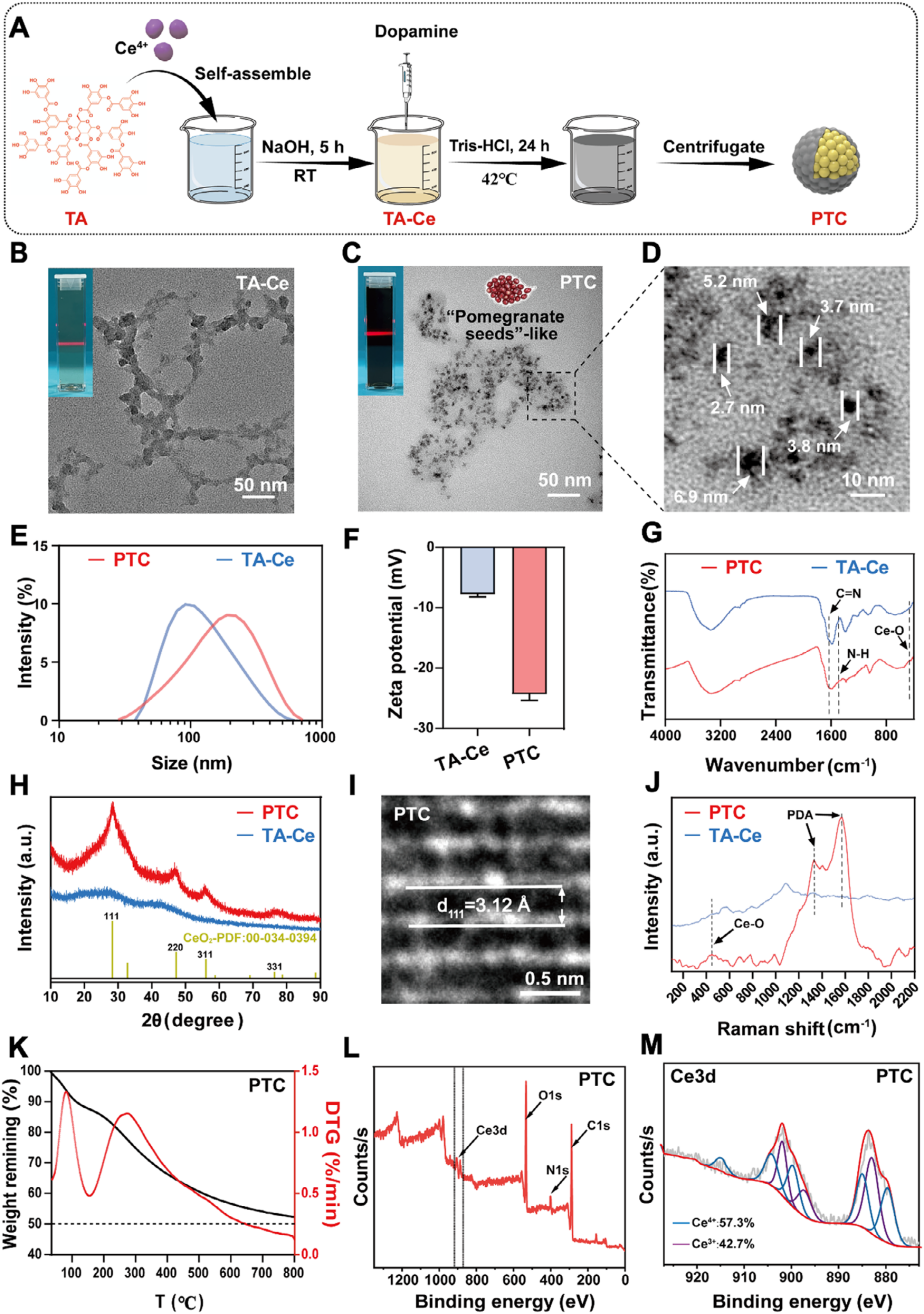
Synthesis and characterization of PTC. A) Synthesis steps of PTC (Created with BioRender.com). TEM images of TA‐Ce B) and PTC C). (Inserted image shows Tyndall effect of TA‐Ce and PTC). D) Partial enlarged view of C). E) Hydrodynamic particle size distribution of TA‐Ce and PTC. F) Zeta potentials of TA‐Ce and PTC. G) FT‐IR spectra of TA‐Ce and PTC. H) XRD patterns of TA‐Ce and PTC. I) HRTEM images of PTC. J) Raman spectra of TA‐Ce and PTC. K) TGA of PTC. L) Full XPS spectrum of PTC. M) Peak separation analysis of XPS spectrum of PTC to determine valence states of Ce.

The PDA‐TA‐Ce nanozymes (PTC) was synthesized by a TA‐mediated one‐pot method,^[^ ^31^ ^]^ followed by surface modification via dopamine polymerization (**Figure** **1**A). Transmission electron microscopy (TEM) images showed that TA‐Ce precursors formed chain‐like nanostructures with obvious Tyndall effect (Figure 1B), confirming their colloidal nature. Upon PDA coating, the resulting PTC adopted a “pomegranate seed”‐like morphology (Figure 1C), composed of densely packed individual particles ranging from 2.7 to 6.8 nm in diameter (Figure 1D). Dynamic light scattering analysis demonstrated an increase in hydrodynamic size from 121.4 ± 0.9 nm for TA‐Ce to 147.6 ± 0.8 nm for PTC (Figure 1E), which was consistent with successful PDA encapsulation while maintaining excellent colloidal stability over 21 days (Figure S1, Supporting Information). Additionally, the polydispersion index (PDI) value increased from 0.251 ± 0.006 of TA‐Ce to 0.273 ± 0.001 of PDA (Figure S2, Supporting Information), and the surface charge of PTC (‐24.2 ± 1.1 mV) was more negative than that of TA‐Ce (‐7.7 ± 0.5 mV) (Figure 1F), indicating enhanced electrostatic stabilization imparted by the PDA coating.

The characteristic peaks at 1497 and 1612 cm^−1^ in the Fourier‐transform infrared (FT‐IR) spectrum of PTC were attributed to the N─H and C═N vibration stretching of PDA, respectively (Figure 1G),^[^ ^32^ ^]^ confirming the presence of PDA in PTC. X‐ray diffraction (XRD) analysis showed while the TA‐Ce exhibited an amorphous structure,^[^ ^33^ ^]^ PTC demonstrated well‐defined crystalline features matching standard CeO_2_ patterns (PDF: 00‐034‐0394) (Figure 1H). High‐resolution transmission electron microscopy (HRTEM) revealed that PTC displayed distinct lattice stripes with a spacing of 3.12 Å (Figure 1I), which is corresponding to the (111) plane of CeO_2_ (Figure 1H). The reason why PDA coating process promoted the crystal structure change of CeO_2_ is that electron transfer and Redox reactions occur during the polymerization of dopamine monomer, providing local energy for the ordered arrangement of cerium ions. Furthermore, OH^−^ will quickly reacts with cerium ions in the alkaline coating environment, and the resulting Ce(OH)_4_ can dehydrate and transform into more stable crystalline CeO_2._^[^ ^34^ ^]^ The Raman spectrum further supported the composite nature of PTC (Figure 1J), showing both symmetric stretching vibration peak of Ce─O at 447 cm^−1^^[^ ^35^ ^]^ and characteristic peaks of PDA at 1328 and 1569 cm^−1^.^[^ ^36^ ^]^ Material composition analysis by thermogravimetric analysis (TGA) indicated that the weight percentage of CeO_2_ in PTC was close to 50% (Figure 1K). The concentration of cerium (Ce) in PTC was 13.7 µg mL^−1^, as determined by inductively coupled plasma mass spectrometry (ICP‐MS). Full spectrum X‐ray photoelectron spectroscopy (XPS) scanning showed the presence of Ce, oxygen (O), nitrogen (N), and carbon (C) elements in PTC (Figure 1L). The relative proportion of Ce^3+^ and Ce^4+^ was close to 1:1 (Figure 1M), which made PTC more suitable for removing ROS and H_2_O_2._^[^ ^33^ ^]^

The PTC nanozymes exhibited remarkable photothermal properties, with solution temperatures reaching 30.9–45.1 °C under NIR irradiation (808 nm, 1 W cm^−2^) in a concentration‐dependent manner (100 µg mL^−1^ Ce achieved 39.7 °C), while maintaining consistent heating performance across multiple power settings (Figure S3, Supporting Information). In short, we synthesized PTC nanozymes with ultra‐small particle size, large specific surface area, and significant photothermal effect by using TA as a template agent coupled with controlled PDA coating, which makes them promising candidates for catalytic and therapeutic applications.

### 2.2 Preparation and Characterization of PTC‐MP Hydrogel

To enable effective delivery of PTC to cartilage joints, we designed an interpenetrating network hydrogel by introducing magnesium ion‐crosslinked sodium alginate (SA‐Mg) within a thermosensitive poloxamer 407 (P407) matrix to encapsulate PTC, termed PTC‐MP hydrogel. For comparison, controls hydrogel including PTC and P407‐SA hydrogel (PTC‐P) and the composite hydrogel of MgCl_2_ and P407‐SA hydrogel (MP) were similarly prepared. Scanning electron microscopy (SEM) images showed that PTC‐MP hydrogel exhibited a dense, spongy mesh‐like microstructure (Figure S4, Supporting Information), which is beneficial for the sustained release of Mg^2+^and PTC.^[^ ^37^ ^]^ Elemental mapping via EDS confirmed that Ce and Mg were evenly distributed in PTC‐MP hydrogel (Figure S5, Supporting Information). The solution‐gel transition of PTC‐MP hydrogel rapidly completed within 5 min at 37 °C (Figure S6A, Supporting Information). PTC‐MP aqueous solution rapidly transformed into a gel state when injected into PBS with a syringe at 37 °C (Figure S6B, Supporting Information), indicating that the excellent injectability of PTC‐MP hydrogel (Figure S6C, Supporting Information), which is adaptable to the irregular articular cavity.

In addition, PTC‐MP hydrogel exhibited excellent bearing capacity, which collapsed only when the strain reached 2.5% (Figure S7A, Supporting Information). Rheological analysis via oscillation frequency (0.1–10 Hz) sweep demonstrated the elastic behavior of the hydrogel with storage modulus (G', 4670–7590 Pa) consistently dominated over its loss modulus (G″, 887–1570 Pa) (Figure S7B, Supporting Information). The excellent mechanical property ensured its compatibility to match joint movements.^[^ ^38^ ^]^

### 2.3 In Vitro and In Vivo mPTT Effects of PTC‐MP Hydrogel

**Figure 2:**
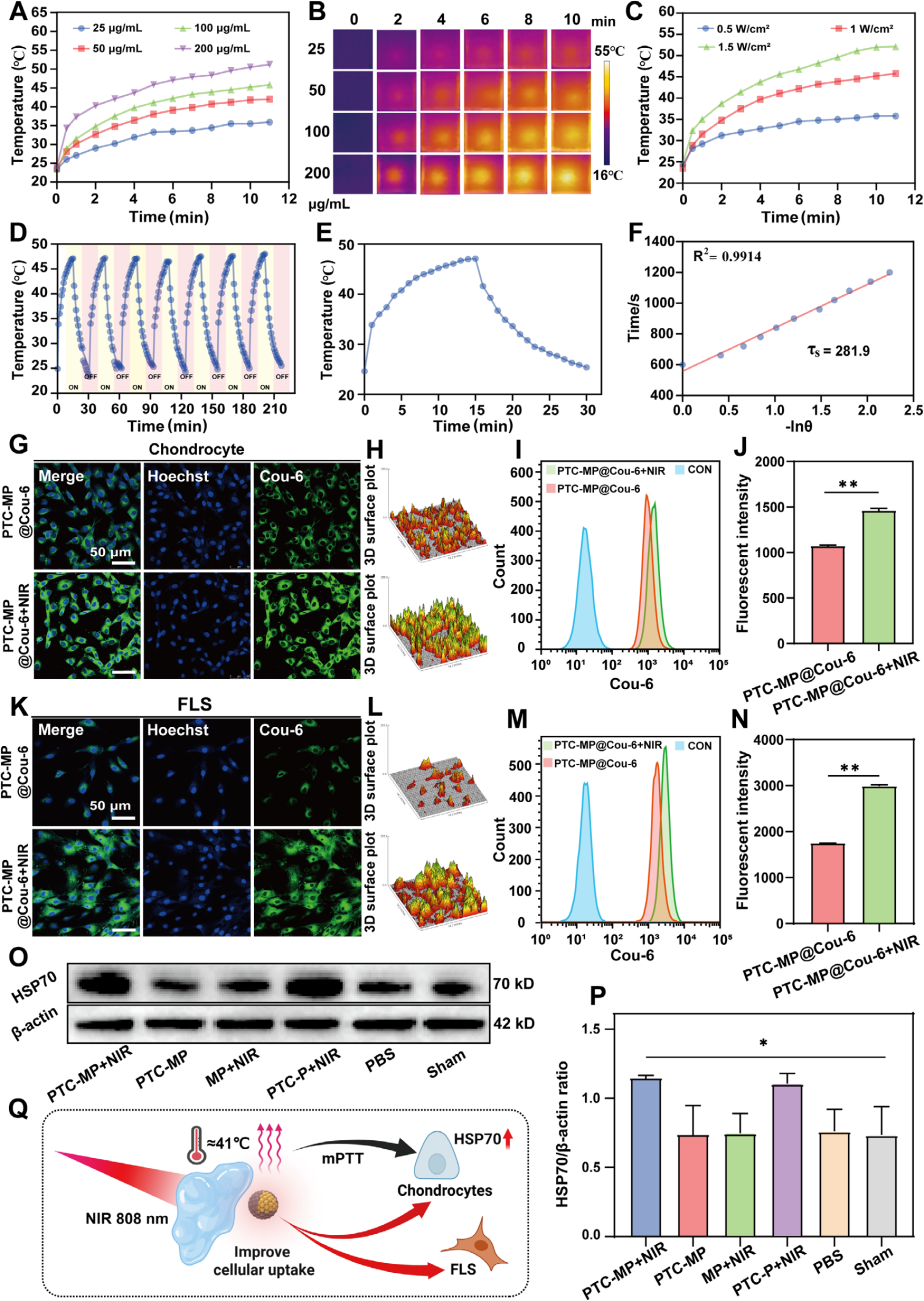
In vitro and in vivo mPTT of PTC‐MP. Temperature curves A) and infrared thermal images B) of PTC‐MP at different Ce concentrations (25, 50, 100, and 200 µg mL^−1^) under NIR irradiation (808 nm, 1.0 W cm^−2^). (C) Temperature curves of PTC‐MP (Ce concentration of 100 µg mL^−1^) under NIR irradiation with different power (0.5, 1.0, and 1.5 W cm^−2^). D) Temperature curves of PTC‐MP under 7 ON/OFF cycles (808 nm, 1.0 W cm^−2^). E) Temperature curve of PTC‐MP (Ce concentration of 100 µg mL^−1^) during laser irradiation and natural cooling process. F) Linear fitting plot of time versus −ln(θ) during the cooling process. Fluorescence images G) and 3D surface plot analysis H) of uptake profiles of PTC‐MP by chondrocytes with or without NIR irradiation. Flow cytometry I) and quantitative analysis J) of cellular uptake of PTC‐MP by chondrocytes with or without NIR irradiation (n=3). Fluorescence images K) and 3D surface plot analysis L) of uptake of PTC‐MP by FLS with or without NIR irradiation. Flow cytometry M) and quantitative analysis N) of cellular uptake of PTC by FLS with or without NIR irradiation (n=3). WB result O) and Semi‐quantitative analysis P) of HSP70 in chondrocytes in different groups (n=3). Q) Schematic illustration of mPTT increasing cellular uptake of PTC and HSP70 expression in chondrocytes (Created with BioRender.com). Data are presented as mean values ± SD. Comparisons were performed by one‐way ANOVA followed by Tukey's multiple comparisons test in P) and unpaired two‐tailed Student's *t*‐test in (J, N). (*p < 0.05, **p < 0.01).

The photothermal capacity of PTC‐MP hydrogel was concentration‐dependent under NIR irradiation (808 nm, 1 W cm^−^^2^) for 5 min, and the temperature increased with increasing PTC concentration (**Figure** **2**A). Infrared thermal imaging showed that the temperature of PTC‐MP hydrogel increased with the increase of Ce concentration (Figure 2B). The photothermal performance of PTC‐MP hydrogel was power‐dependent, and the temperature reached 45.6 °C under 1.5 W cm^−^^2^ laser irradiation for 5 min (Figure 2C). The temperature curve of PTC‐MP hydrogel showed good stability (Figure 2D) after 7 on/off cycles, and its photothermal conversion efficiency (η) was 34.7% (Figure 2E,F). Additionally, the cumulative release rates of Mg^2+^ from PTC‐MP and PTC‐MP+NIR were 43.9 % and 64.5 % on day 5 (Figure S8, Supporting Information), respectively, indicating that NIR promoted the release of Mg^2+^.

In addition, we tested other parameters in the selection of photothermal parameters in vivo. The temperature of the knee joint of rats in the PTC‐MP group was not raised to 40 °C within 1 0 min both under 0.1 and 0.2 W cm^−^^2^ (Figure S9, Supporting Information). However, the temperature of the knee joint of rats exceeded 45 °C in just 2 min when the power reached 1 W cm^−^^2^, which will cause irreversible thermal burns to the animals. Based on the animal ethics and safety, we chose the photothermal parameter of 0.4 W cm^−^^2^. The temperature of the knee joint of rats in PTC‐MP group was raised to ≈41 °C after NIR irradiation with 0.4 W cm^−^^2^ for 2 min, while that of MP group remained at 37 °C after NIR irradiation for 10 min (Figure S10, Supporting Information). These results indicated that PTC‐MP hydrogel had significant mPTT effect in vivo. It has been reported that 40–42 °C is the best temperature range for OA treatment.^[^ ^23^, ^28^ ^]^ Therefore, PTC‐MP hydrogel with Ce concentration of 400 µg mL^−1^ (41.1 °C) was selected for the follow‐up study against DOA in vivo.

### 2.4 Enhancement of PTC‐MP Internalization and HSP70 Expression by NIR

The internalization of PTC in PTC‐MP hydrogel by cells is a prerequisite for its therapeutic effect. Compared with non‐NIR treatment group, the green fluorescence of PTC in chondrocytes (Figure 2G,H) and FLS (Figure 2K,L) after NIR treatment was significantly enhanced, and the uptake of PTC by chondrocytes or FLS was increased by 1.4 times and 1.7 times, respectively (Figure 2I,J, M,N), indicating that NIR irradiation promoted cellular uptake of PTC. We speculate that this effect mainly caused by dopamine‐induced mPTT in the PTC system. Photodynamic therapy mainly generates reactive oxygen species (ROS) without causing an increase in temperature, and our experimental results showed that PTC has no significant photodynamic effect under NIR (Figure S11, Supporting Information). In addition, it has been reported that mPTT can increase the fluidity of cell membranes and enhance the cellular uptake.^[^ ^39^ ^]^ It can also enhance the enzymatic activity of nanozymes and the production of oxygen (Figure S17, Supporting Information). The high level of HSP70 has been proven to inhibit inflammation, reduce apoptosis^[^ ^40^ ^]^ and resist OA.^[^ ^26^ ^]^ Results showed that the expression of HSP70 in chondrocytes was increased by mPTT of PTC‐MP hydrogel under NIR irradiation (Figure 2O–Q), which is beneficial to inhibit OA inflammation.

In addition, the biocompatibility of PTC‐MP hydrogel is a prerequisite for its application in vivo. Cell Counting Kit‐8 (CCK8) was used to detect the toxicity of PTC and Mg^2+^ to chondrocytes and FLS. The survival rate of both cells was 90% when the concentration of Ce in PTC was 100 µg mL^−1^ (Figure S12A,B, Supporting Information). While the cell survival rate dropped to less than 80% when Ce concentration reached 200 µg mL^−1^. Therefore, we chose PTC with Ce concentration of 100 µg mL^−1^ for subsequent experiment. Notably, the toxicity of Mg^2+^ to both cells was negligible (Figure S12C,D, Supporting Information). To evaluate its hemolysis, PTC containing Ce concentrations of 25, 50, 100, and 200 µg mL^−1^ were incubated with red blood cells for 24 h, respectively (Figure S13, Supporting Information). Our results showed that PTC had no obvious damage to red blood cells, even at the highest Ce concentration of 200 µg mL^−1^, indicating that PTC had good blood compatibility.

### 2.5 Effect and Mechanism of PTC Inhibiting the Formation of AGEs

**Figure 3:**
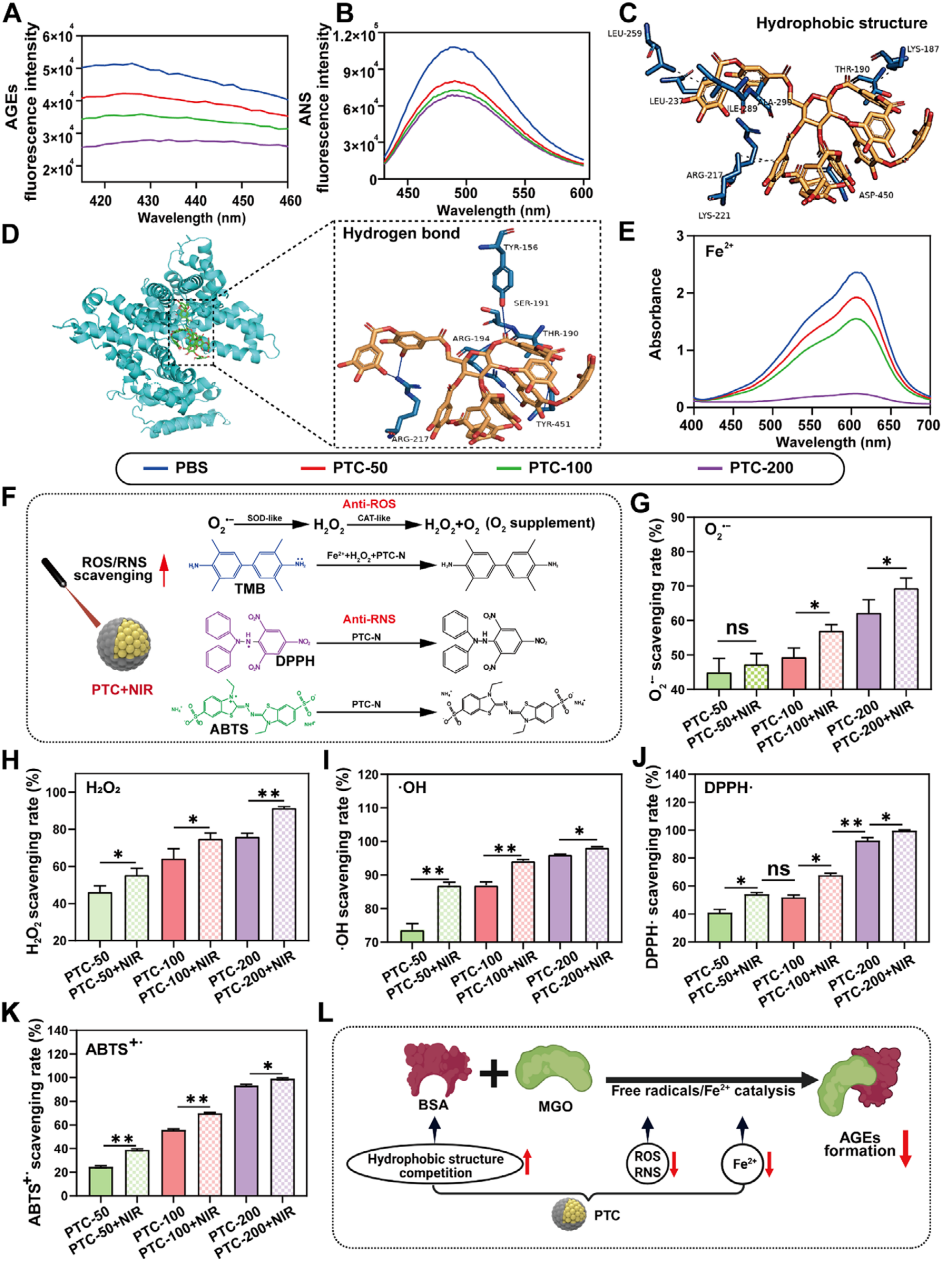
Effect and mechanism of PTC in inhibiting AGEs formation In vitro. A) Fluorescence spectra of AGEs in the presence of different concentration of PTC. B) Fluorescence spectra of ANS binding to BSA. Molecular docking results of TA with hydrophobic residues C) and hydrogen bond D) of BSA. E) UV‐vis absorbance spectra of Fe^2+^ chelation ability by PTC at different concentration. F) Schematic illustration of PTC‐N for ROS/RNS scavenging and O_2_ supplement. Quantitative analysis of O_2_^•−^ G) and H_2_O_2_ H) scavenging rates of PTC at different concentration under NIR irradiation (808 nm, 1 W cm^−2^) (n=4). Quantitative analysis of ·OH I), DPPH·J) and ABTS^+·^ K) scavenging rates of PTC at different concentration under NIR or non‐NIR (n=3). L) Mechanism of PTC inhibiting AGEs formation (Created with BioRender.com). Data are presented as mean values ± SD. Comparisons were performed by one‐way ANOVA followed by Tukey's multiple comparisons test in G, H, I, J, K). (*p < 0.05, **p < 0.01, ns means no statistical significance).

An experimental model of pyruvate‐bovine serum protein (MGO‐BSA) glycation was constructed to explore the inhibition of AGEs by PTC and its mechanism In vitro. The results showed that the fluorescence intensity of AGEs decreased with the increase of PTC concentration (**Figure** **3**A), and the inhibition rate of AGEs reached 43.6% (Figure S14, Supporting Information) when the concentration of Ce was 200 µg mL^−1^ (PTC‐200), indicating that PTC effectively inhibited the formation of AGEs.

It has been reported that the binding site of α‐dicarbonyl compound (α‐DC) to bovine serum albumin (BSA) is located in its hydrophobic region,^[^ ^41^ ^]^ which can produce fluorescence by binding with 8‐aniline‐1‐naphthalene sulfonic acid (ANS) dye. Calculated by ANS fluorescence staining, the inhibition rate of PTC‐200 on the hydrophobic structure of BSA was 36.4% (Figure 3B; Figure S15, Supporting Information). Molecular docking simulation results showed that there were hydrophobic interactions between TA and amino residues of BSA, such as Leu237, Leu259 and Ile289 (Figure 3C), and hydrogen bond interactions with amino residues such as Tyr156, Tyr451, and Ser191 (Figure 3D). These results indicated that the binding of TA to BSA was mainly driven by hydrogen bonding and hydrophobic interactions, which facilitate PTC to inhibit protein glycosylation of AGEs by competing with α‐DC for hydrophobic sites.

Fe^2+^ has been reported to accelerate the process of glycosylation.^[^ ^42^ ^]^ Our results showed that the chelation of Fe^2+^ by PTC was concentration‐dependent (Figure 3E). The inhibition rate of PTC‐200 on Fe^2+^ was 89.7% (Figure S16, Supporting Information), which is beneficial for PTC to inhibit the glycosylation of AGEs by chelating Fe^2+^.

Free radicals in diabetic patients include ROS and reactive nitrogen species (RNS), which can promote the generation of AGEs.^[^ ^43^ ^]^ The ability of PTC to remove these harmful free radicals under NIR irradiation was evaluated (Figure 3F). Our results showed that the scavenging rate of superoxide anion radical (O_2_^·−^) by PTC increased from 45.0% to 69.4% when the concentration of Ce in PTC increased from 50 to 200 µg mL^−1^ (Figure 3G). A similar trend was also found in PTC decomposition of H_2_O_2_ (Figure 3H), which was attributed to the CAT‐like enzyme activity of PTC (Figure S17A, Supporting Information). Quantitative results showed that the dissolved oxygen content of PTC‐200 under NIR irradiation (PTC‐200+NIR) was significantly higher than that of PTC‐200 group (Figure S17B, Supporting Information), indicating that PTC is beneficial for alleviating hypoxia of DOA. The 3,3',5,5' ‐tetramethylbenzidine (TMB) method was used to evaluate the scavenging ability of PTC on hydroxyl radical (·OH), and our results showed that the ultraviolet (UV) absorption value of TMB decreased in a concentration‐dependent manner after PTC treatment (Figure S18A, Supporting Information). PTC‐200+NIR scavenged 98% of the ·OH radicals within 2 h (Figure 3I), indicating that PTC efficiently removed ROS, which is beneficial for reducing the generation of AGEs. Additionally, there were no significant difference in the SOD and CAT activities of PTC nanozyme between day 1 and day 10 under NIR or non‐NIR conditions (Figure S19, Supporting Information), indicating that PTC has good enzyme activity stability.

The scavenging results of 2, 2‐diphenyl‐1‐pyridinium (DPPH·) and 2,2 ′‐diazo‐bis ‐(3‐ethylbenzothiazolin‐6‐sulfonic acid) (ABTS^+·^) by PTC showed that the absorbance of DPPH· at 517 nm and ABTS^+·^ at 415 nm were significantly reduced after PTC‐200+NIR treatment, compared with phosphate buffer saline (PBS) group (Figure S18B,C, Supporting Information). The scavenging rates of PTC to DPPH· and ABTS^+·^ were close to 100% within 30 min (Figure 3J,K), indicating that PTC had excellent RNS clearance ability under NIR irradiation.

In short, the mechanism by which PTC inhibits AGEs mainly involves competing with the hydrophobic structure of binding proteins, chelating metal ions, and scavenging free radicals (Figure 3L).

### 2.6 Protective Effect of PTC‐MP with mPTT on Cartilage

**Figure 4:**
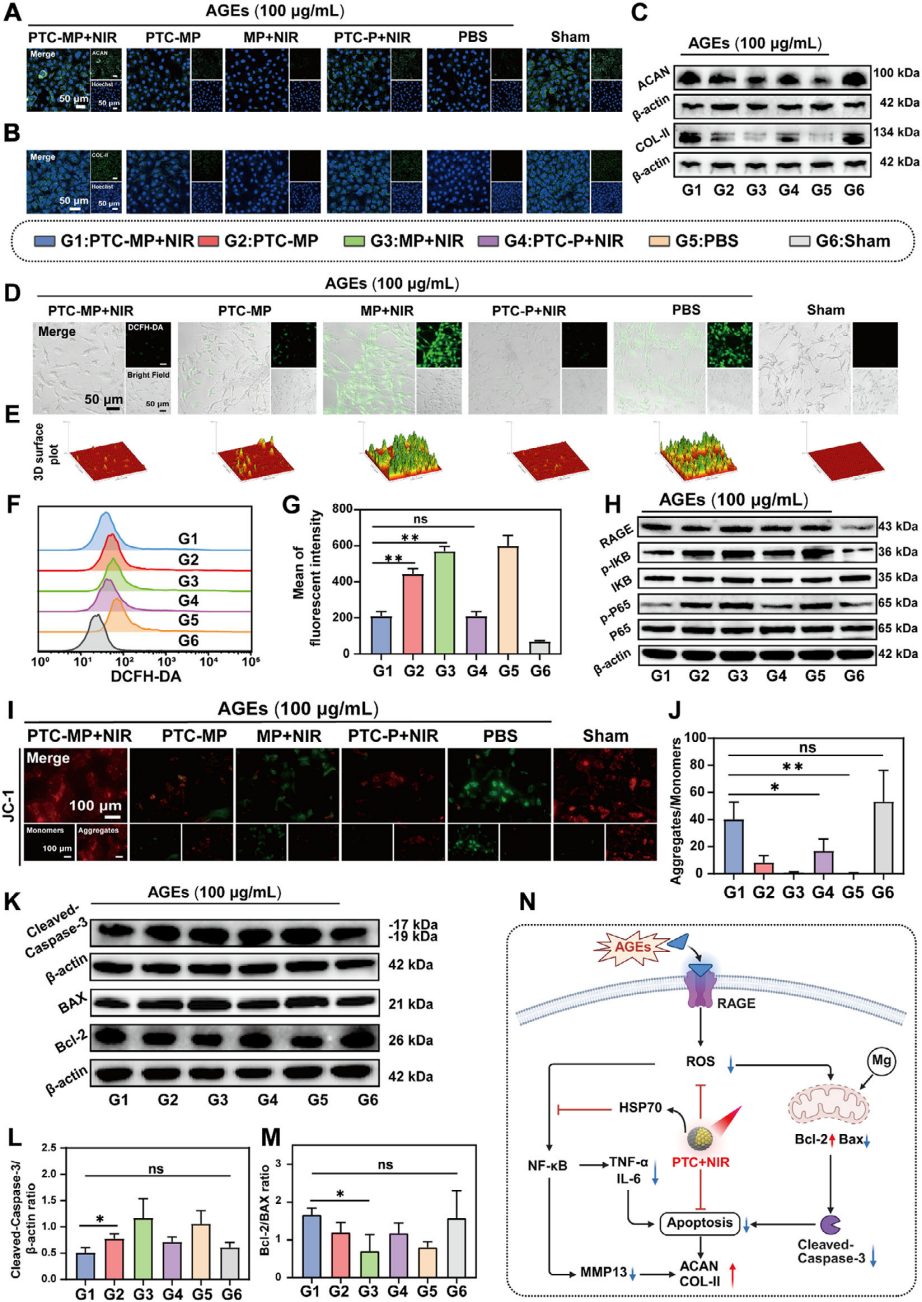
Protective effect and mechanisms of PTC‐MP hydrogel under mPTT on cartilage. Fluorescence images of COL‐II A) and ACAN B) expression in chondrocytes treated with different groups. C) WB results of COL‐II and ACAN expression in chondrocytes treated with different groups. Fluorescence images D) and 3D surface plot analysis (E) of intracellular ROS levels in chondrocytes incubated with different groups. Flow cytometry F) and quantitative analysis G) of intracellular ROS levels in chondrocytes treated with different groups (n=3). H) WB results of RAGE expression and NF‐𝜅B pathways (p‐P65, P65, p‐IKB and IKB). Fluorescence images (I) and semi‐quantitative analysis J) of chondrocytes stained with JC‐1 in different groups (n=3). K) WB results of apoptosis‐related proteins (cleaved‐caspase‐3, Bcl‐2 and BAX) in different groups. Semi‐quantitative analysis of cleaved‐caspase‐3 L) (n=6) and Bcl‐2/BAX (M) (n=5) in different groups. N) Diagram of the mechanism of chondrocyte protection (Created with BioRender.com). Data are presented as mean values ± SD. Comparisons were performed by one‐way ANOVA followed by Tukey's multiple comparisons test in (G, J, L, M). (*p < 0.05, **p < 0.01, ns means no statistical significance).

The chondrocyte specific markers collagen‐II (COL‐II) and aggrecan (ACAN) proteins have been reported to decrease after chondrocyte injury.^[^ ^44^ ^]^ Fluorescence staining results showed that the fluorescence intensity of COL‐II and ACAN in PTC‐MP hydrogel under NIR irradiation (PTC‐MP+NIR) group was higher than that of PBS treated group (**Figure** **4**A,B), indicating that PTC‐MP+NIR effectively protected cells from the damage of AGEs. Fluorescence semi‐quantitative statistical analysis showed that the protective effect of PTC‐MP+NIR was significantly improved compared with that of PBS group (Figures S20,S21, Supporting Information). Western Blotting (WB) results also showed that PTC‐MP+NIR upregulated the expression of COL‐II and ACAN in chondrocyte in comparison with PBS group (Figure 4C; Figures S22,S23, Supporting Information). These results showed that PTC‐MP+NIR significantly restored chondrocytes homeostasis.

### 2.7 Protective Mechanism of PTC‐MP with mPTT on Cartilage

PTC‐MP+NIR treatment enhanced cellular resistance to AGEs‐induced oxidative/inflammatory stress, ameliorated mitochondrial dysfunction, and promoted chondrocyte synthesis of ACAN and COL‐II. Our results showed that stimulating chondrocytes with AGEs caused an increase in receptor of advanced glycation end products (RAGE) expression (Figure 4H; Figure S24, Supporting Information). Fluorescence images and flow cytometry quantitative data showed that the ROS level in chondrocytes was increased after AGE (100 µg/mL) stimulation for 24 h, while PTC‐MP+NIR significantly decreased the level of ROS (Figure 4D–G). The clearance of ROS in PTC‐MP+NIR group was higher than that in PTC‐MP group, which may be the high expression of HSP 70 induced by mPTT inhibits the production of ROS.^[^ ^45^ ^]^ In addition, the difference between PTC‐MP hydrogel and commercially available Sodium Hyaluronate (HA) gel was also evaluated. Fluorescence images and semi‐quantitative data showed that the green fluorescence signal of HA gel group was higher than that of PTC‐MP (Figures S25,S26, Supporting Information), indicating that PTC‐MP effectively reduced the AGEs‐induced ROS. WB results showed that PTC‐MP+NIR effectively inhibited the AGEs‐induced activation of Nuclear Factor kappa‐light‐chain‐enhancer of activated B cells (NF‐𝜅B) pathway (Figure 4H; Figures S27,S28, Supporting Information). The expression levels of inflammatory cytokines (IL‐6, TNF‐α) and MMP‐13 were decreased (Figure S29, Supporting Information), indicating that PTC‐MP+NIR effectively reduced the secretion of inflammatory factors by inhibiting AGEs induced NF‐𝜅B pathway.

The protective effect of PTC‐MP+NIR on mitochondria was evaluated by JC‐1 staining and Mito‐Tracker Red CMXRos staining. JC‐1 staining results showed that the red fluorescence intensity in PTC‐MP+NIR group was higher than that in PTC‐P hydrogel under NIR radiation (PTC‐P+NIR) group (Figure 4I,J), indicating that Mg^2+^ enhanced the protective effect on mitochondria.^[^ ^46^ ^]^ It has been reported that Mg^2^⁺ is an essential cofactor for enzymes related to ATP generation and the Mg‐ATP complex,^[^ ^47^ ^]^ which plays an important role in the process of oxidative phosphorylation. Adequate supply of Mg^2^⁺ helps ATP production in mitochondria, thereby supporting the normal function of cells. In addition, Mg^2^⁺ has been found to have significant anti‐inflammatory and antioxidant effects,^[^ ^48^ ^]^ and can alleviate chondrocyte death in osteoarthritis models.^[^ ^49^ ^]^ Mito‐Tracker Red CMXRos staining results showed that red fluorescence intensity in PTC‐MP+NIR group was higher than that in PBS group (Figures S30,S31, Supporting Information), indicating that PTC‐MP+NIR effectively protected mitochondrial function. Additionally, WB semi‐quantitative analysis showed that PTC‐MP+NIR downregulated the protein expression of BCl2‐associated X protein (BAX) and Cleaved caspase‐3, and upregulated expression of B‐cell lymphoma 2 (BCL‐2), indicated that PTC‐MP+NIR effectively inhibited mitochondria‐mediated apoptosis triggered by AGEs (Figure 4K–M). These results indicated that PTC‐MP hydrogel effectively restored cartilage homeostasis through anti‐oxidation, anti‐inflammatory, inhibition of NF‐𝜅B inflammatory pathway and protection of mitochondria in diabetes microenvironment simulated by AGEs In vitro (Figure 4N).

### 2.8 Effect of PTC‐MP with mPTT on HA Secretion and its Mechanism

**Figure 5:**
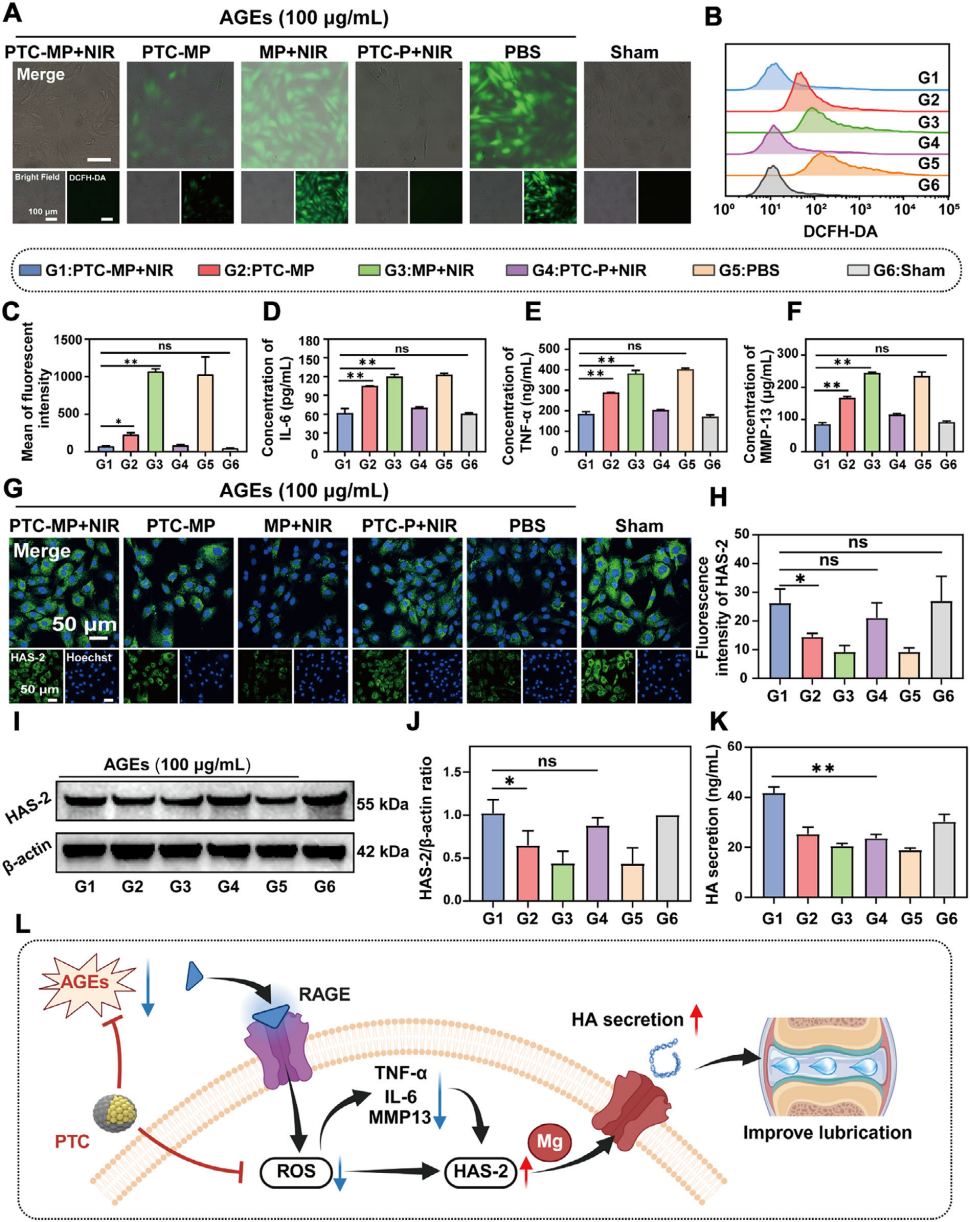
Promotion and mechanism of HA secretion from FLS by PTC‐MP+NIR. Fluorescence images A) and flow cytometry B) of intracellular ROS levels in FLS treated with different groups. C) Semi‐quantitative analysis of intracellular ROS levels of FLS treated with different groups (n=3). IL‐6 D), TNF‐α E), and MMP13 F) expression in FLS by ELISA assay quantification in different groups (n=3). Fluorescence images G) and semi‐quantitative analysis H) of HAS‐2 expression in FLS treated with different groups (n=6). WB results I) and semi‐quantitative analysis J) of HAS‐2 expression in FLS (n=3). K) HA expression in FLS by ELISA assay quantification (n=3). L) Diagram of mechanism of promoting HA secretion from FLS (Created with BioRender.com). Data are presented as mean values ± SD. Comparisons were performed by one‐way ANOVA followed by Tukey's multiple comparisons test in C–F, H, J, K). (*p < 0.05, **p < 0.01, ns means no statistical significance).

Hyaluronic acid (HA), a major synovial fluid component primarily synthesized by fibroblast‐like synoviocytes (FLS), provides essential joint lubrication.^[^ ^50^ ^]^ ROS fluorescence staining and flow cytometry results showed that the green fluorescence intensity of FLS treated by PTC‐MP+NIR groups was significantly decreased compared with PBS group, and had no significant difference compared with Sham group (**Figure** **5**A–C), indicating that PTC‐MP+NIR significantly cleared ROS from FLS. The levels of IL‐6, TNF‐α and MMP‐13 were significantly reduced in the cell supernatant of FLS treated with PTC‐MP+NIR (Figure 5D–F), indicating that PTC‐MP+NIR significantly protected FLS and reduced the secretion of inflammatory factors.

Endogenous HA is mainly synthesized by HAS‐2 in FLS.^[^ ^51^ ^]^ Consequently, preserving HAS‐2 protein function and enhancing endogenous HA secretion from FLS represent a viable therapeutic approach to counteract lubrication deficiency in osteoarthritis.^[^ ^52^ ^]^ Fluorescence staining results showed that the green fluorescence intensity of HAS‐2 in PTC‐MP+NIR group was significantly higher than that in PBS group, and was close to that in Sham group (Figure 5G,H). Quantitative results of WB showed that the HAS‐2 expression in PTC‐MP+NIR group was higher than that in PBS groups (Figure 5I,J), indicating that PTC‐MP+NIR protected HAS‐2 from AGEs damage. More importantly, ELISA results showed that the content of HA in the supernatant of FLS in PTC‐MP+NIR group was significantly higher than that in PTC‐P+NIR group (*p* < 0.01), indicating that Mg^2+^ effectively promoted HA secretion (Figure 5K). It has been reported that Mg^2^⁺ can directly participate in the synthesis of HA from HAS‐2, which can enhance HA production.^[^ ^53^ ^]^ Therefore, PTC‐MP+NIR protected FLS through anti‐inflammatory and antioxidant effects, and effectively enhanced cartilage lubrication by stimulating HA secretion (Figure 5L).

### 2.9 Therapeutic Effect of PTC‐MP with mPTT Against DOA In Vivo

**Figure 6:**
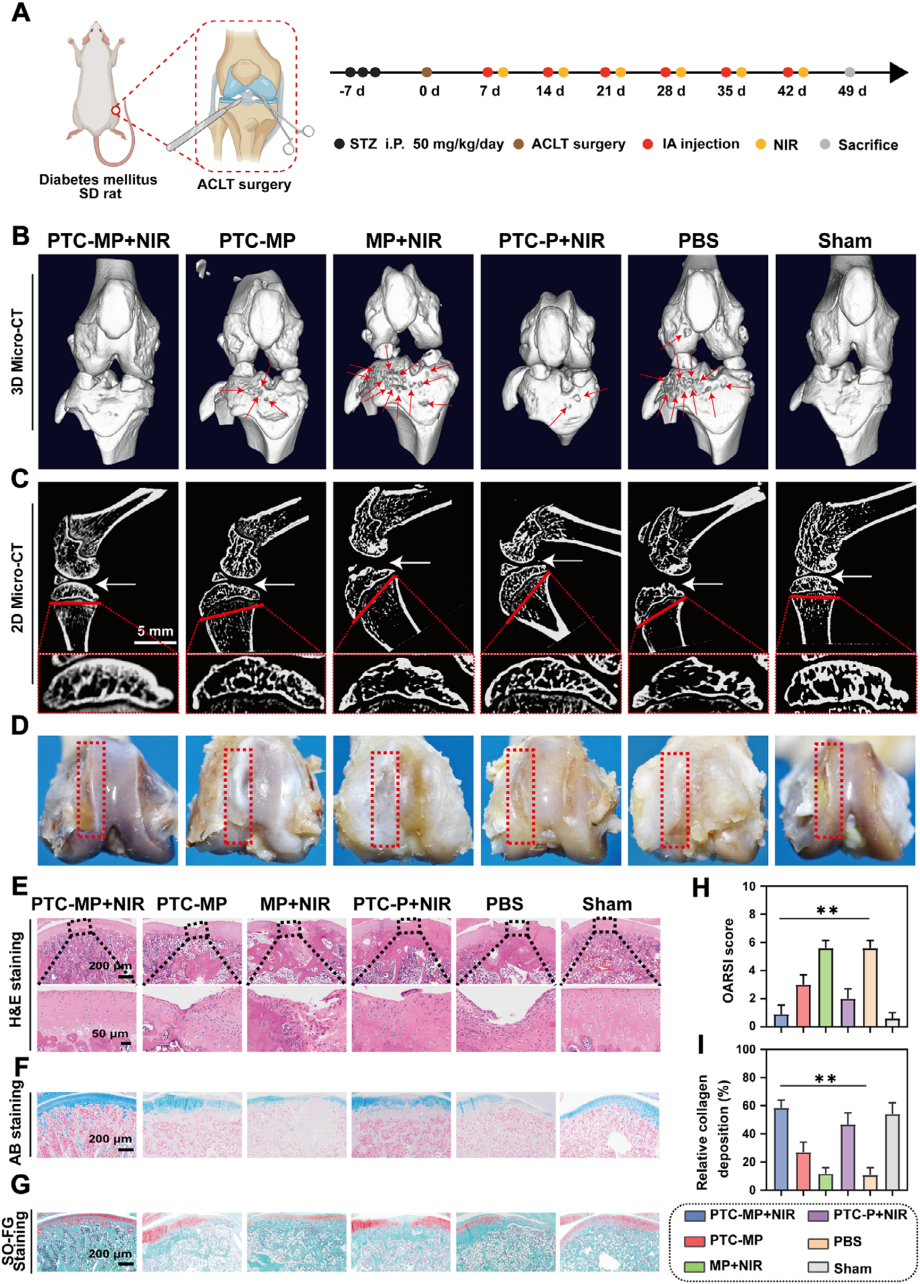
Therapeutic effect of PTC‐MP under mPTT in a DOA model. A) Schematic diagram of DOA model establishment and treatment method (NIR, 808 nm, 0.4 W cm^−2^, 10 min) (Created with BioRender.com). B) Representative micro‐CT scanning and reconstruction of knee joints after different treatments for 7 weeks. C) Representative micro‐CT images of subchondral bone. D) Photos of the joint after different treatments for 7 weeks. H&E staining E), AB staining F) and SO‐FG staining G) of cartilage sections after different treatments for 7 weeks. OARSI score (n=6) H) and relative collagen deposition (n=6) I) of joints after different treatments for 7 weeks. Data are presented as mean values ± SD. Comparisons were performed by one‐way ANOVA followed by Tukey's multiple comparisons test in H, I). (*p < 0.05, **p < 0.01, ns means no statistical significance).

The diabetic model of SD rats was induced by streptozotocin (STZ), and then a DOA model was constructed by anterior cruciate ligament transection (ACLT).^[^ ^54^ ^]^ DOA rats were injected with different formulations in the knee joint in situ and were exposed to 808 nm laser (0.4 W cm^−^^2^; 10 min; once a week) (**Figure** **6**A). During the whole treatment period, the blood glucose of SD rats was higher than 16.7 mmol L^−1^ (Figure S32, Supporting Information).

Next, the therapeutic effect of PTC‐MP hydrogel with mPTT was explored in vivo. Micro‐CT results showed that the surface of subchondral bone in Sham group was smooth and complete, while the surface of subchondral bone in MP under NIR radiation (MP+NIR) and PBS groups showed obvious bone erosion, fractures, and cartilage degradation (Figure 6B). There were no significant cracks and cartilage degradation on the joint surface in PTC‐MP+NIR group, which had no significant difference from that in Sham group. While the joint space was larger in MP+NIR and PBS groups, and their subchondral bone injury was obvious (Figure 6C). Photos of the joint showed that the cartilage surface in PTC‐MP+NIR group had no obvious wear with intact morphology, and there was no degeneration of cartilage. However, normal cartilage morphology was not observed in PBS group and MP+NIR group, and their cartilage deformation was severe (Figure 6D). These results indicated that PTC‐MP hydrogel effectively inhibited the development of DOA in vivo.

The histopathological changes of articular cartilage were investigated by Hematoxylin and eosin (H&E) staining, Alcian blue (AB) staining and safranin O‐Fast Green (SO‐FG) staining. The cartilage surface erosion was severe, and fibrous tissue hyperplasia was obvious in PBS group and MP+NIR group, accompanied by a large number of inflammatory cell infiltration (Figure 6E). While the cartilage surface damage in PTC‐MP+NIR group showed obvious recovery, with continuous tide lines and relatively intact cartilage structure. The Osteoarthritis Research Society International (OARSI) scores in PBS group was significantly higher than that in PTC‐MP+NIR group (Figure 6H), indicating that PTC‐MP+NIR effectively inhibited cartilage destruction. Results of AB and SO‐FG staining showed noticeable loss of cartilage extracellular matrix in Sham group (Figure 6F), indicating that AGEs accumulation led to the destruction of cartilage matrix.^[^ ^55^ ^]^ The cartilage matrix in PTC‐MP+NIR group was uniformly blue with clear tide lines. However, the blue stains of cartilage tissue became lighter and the tide line became unclear in PBS group, indicating that PTC‐MP hydrogel effectively maintained collagen deposition (Figure 6G, l) and cartilage thickness (Figure S33, Supporting Information). These results indicated that PTC‐MP+NIR effectively inhibited the progression of DOA.

**Figure 7:**
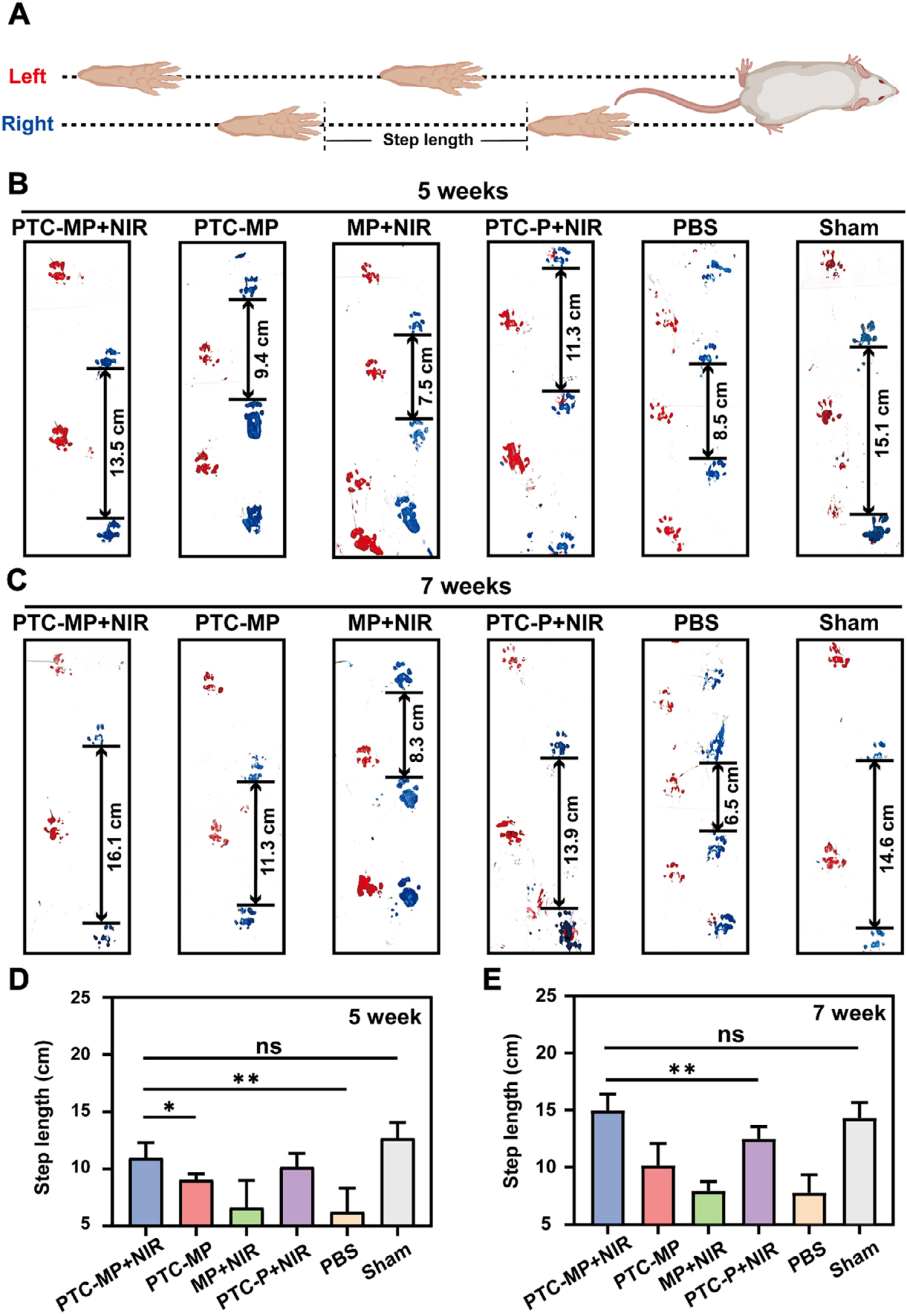
Behavior experiment of rats in a DOA model. A) Schematic diagram of footprint analysis behavior experiment (Created with BioRender.com). Footprints of rats after 5 weeks B) and 7 weeks C) of treatment. Quantitative analysis of step length in different groups after 5 weeks D) and 7 weeks E) (n=6). Data are presented as mean values ± SD. Comparisons were performed by one‐way ANOVA followed by Tukey's multiple comparisons test in D, E). (*p < 0.05, **p < 0.01, ns means no statistical significance).

Additionally, we labeled the left and right hind feet of rats with red and blue dyes, respectively, to further evaluate the therapeutic effect by footprint analysis (**Figure** **7**A). The step lengths of rats in PTC‐MP+NIR, PTC‐P+NIR, and PTC‐MP groups were 12.3 ± 1.1, 8.8 ± 0.6, and 10.2 ± 1.1 cm, respectively, after 5 weeks of experiment. Compared with Sham group, PTC‐MP+NIR group showed no significant difference in step length (Figure 7B,D). While the step length of rats in MP+NIR and PBS groups were only 5.9 ± 1.7 and 6.3 ± 2.0 cm, indicating that the cartilage damage was serious and affected the normal walking function of rats. It was noteworthy that the step length of rats in PTC‐MP+NIR group reached 16.3 ± 0.2 cm at week 7, which was significantly increased compared with 12.3 ± 1.1 cm at week 5 (Figure 7C,E). These results indicated that PTC‐MP+NIR effectively improved the gait of rats.

The retention of hydrogel in vivo was investigated by live animal imaging (Figure S34, Supporting Information). Our results showed that most of PTC‐MP hydrogel was still concentrated in the joint cavity after injection for 7 days, and the fluorescence signal lasted until the 14th day, indicating that PTC‐MP hydrogel had a good retention ability in the joint cavity.^[^ ^56^ ^]^ The mass of the PTC‐MP hydrogel decreased over time in the simulated synovial fluid (Figure S35A, Supporting Information), which was consistent with the trend of weakened fluorescence in vivo (Figure S35B, Supporting Information). Furthermore, due to the avascular nature of cartilage tissue, substances injected into the synovial fluid usually circulate locally and are difficult to enter the systemic circulation.^[^ ^57^ ^]^ Eventually, they will be cleared through lymphatic drainage or phagocytosis by synovial macrophages.^[^ ^58^ ^]^ Our experimental results showed that no fluorescence signal was observed in explanted major organs (heart, liver, spleen, lungs, kidneys) on day 7 (Figure S36, Supporting Information), indicating that cerium dioxide nanoparticles did not accumulate in the animals and had good safety.

It has been reported that cerium dioxide nanoparticles exhibit good biocompatibility in biological systems, and they can be gradually degraded and metabolized without causing long‐term accumulation.^[^ ^59^ ^]^ The safety of PTC‐MP hydrogel in major organs was investigated in vivo. H&E staining images of major organs in DOA rats showed that no obvious organ damage was found in the heart, spleen, lungs and kidneys in all groups (Figure S37, Supporting Information), indicating that PTC‐MP hydrogel had good biosafety in vivo.

### 2.10 Therapeutic Mechanism of PTC‐MP with mPTT Against DOA In Vivo

**Figure 8:**
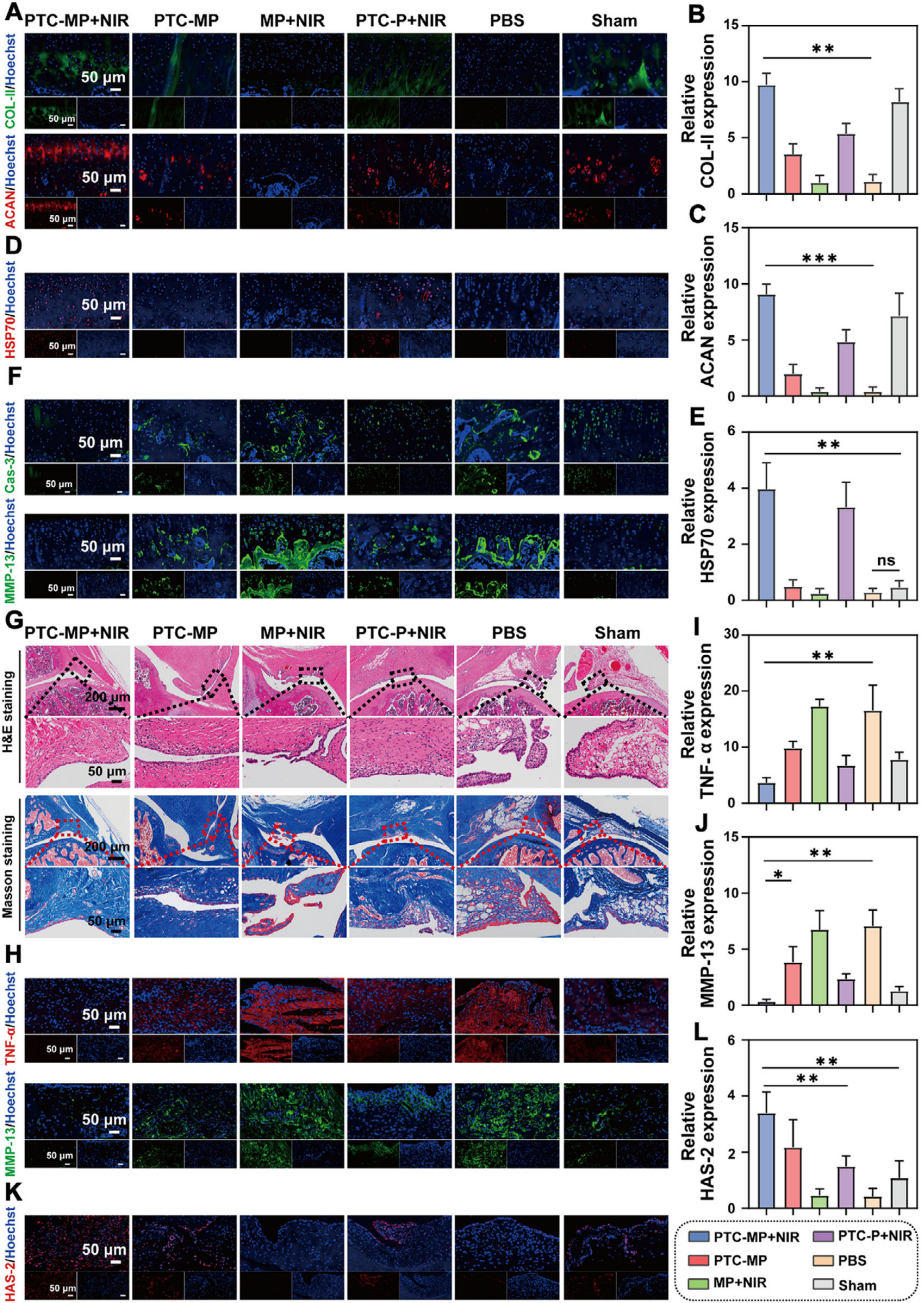
Therapeutic mechanism of PTC‐MP under mPTT in a DOA model after 7 weeks of treatment. A) Immunofluorescence images of COL‐II and ACAN in cartilage sections. Semi‐quantitative analysis of COL‐II B) and ACAN C) in cartilage sections (n=6). Immunofluorescence images D) and semi‐quantitative analysis E) of HSP‐70 in cartilage sections (n=6). F) Immunofluorescence images of caspase‐3 (Cas‐3) and MMP‐13 expression level in cartilage sections. G) H&E staining and Masson staining of synovial tissue sections. H) Immunofluorescence images of TNF‐α and MMP‐13 in synovial tissue sections. Semi‐quantitative analysis of TNF‐α I) and MMP‐13 J) in synovial tissue sections (n=6). Immunofluorescence images K) and semi‐quantitative analysis L) of HAS‐2 in synovial tissue sections (n=6). Data are presented as mean values ± SD. Comparisons were performed by one‐way ANOVA followed by Tukey's multiple comparisons test in B, C, E, I, J, L). (*p < 0.05, **p < 0.01, ***p < 0.001, ns means no statistical significance).

The expression of COL‐2 and ACAN will decrease during cartilage damage.^[^ ^60^ ^]^ Our results of immunofluorescence staining showed that the expressions of COL‐2 and ACAN in PTC‐MP+NIR group were significantly higher than those in PBS group (**Figure** **8**A–C). Immunohistochemical staining results showed that AGEs expression in cartilage tissue in PBS group was higher than that of PTC‐MP+NIR (Figure S38, Supporting Information), indicating that PTC‐MP+NIR effectively reduced the accumulation of AGEs in cartilage tissue. It has been reported that the expression of HSP70 is suppressed in tissues,^[^ ^25^ ^]^ and the levels of inflammatory factors (caspase‐3 and MMP‐13)^[^ ^61^, ^62^ ^]^ are upregulated in diabetic models, which will accelerate the process of DOA. Our immunofluorescence staining data showed that the expression of HSP70 was inhibited (Figure 8D,E) but the expressions of caspase‐3 and MMP‐13 were increased in Sham group (Figure 8F). While the expression of HSP70 in the cartilage tissues of PTC‐MP+NIR and PTC+NIR groups was significantly increased, and the expressions of caspase‐3 and MMP‐13 were decreased (Figures S39,S40, Supporting Information). These results indicated that PTC‐MP hydrogel had a protective effect on cartilage injury in vivo.

To investigate the protective effect of PTC‐MP hydrogel on synovium tissue, H&E and Masson staining were used for histological analysis. A large number of inflammatory cell infiltration and tissue fibrosis were observed in the synovial tissues of PBS and MP+NIR groups (Figure 8G). While the inflammatory cell infiltration was significantly reduced and the tissue morphology returned to normal after treatment with PTC‐MP+NIR. Immunohistochemical staining results showed that AGEs expression in synovial tissue in PBS group was higher than that of PTC‐MP+NIR (Figure S41, Supporting Information), indicating that PTC‐MP+NIR effectively reduced the accumulation of AGEs in synovial tissue. Studies have shown that the expression of TNF‐α and MMP13 is positively correlated with synovial tissue inflammation.^[^ ^63^ ^]^ Our immunofluorescence staining data showed that the expressions of TNF‐α and MMP13 in synovial tissue were significantly decreased after treatment with PTC‐MP+NIR (Figure 8H–J). These results indicated that PTC‐MP hydrogel effectively inhibited the inflammation in synovial tissue under mPTT. In addition, the expression of HAS‐2 in synovial tissue can promote the secretion of endogenous HA.^[^ ^51^ ^]^ Our fluorescence staining results showed that the fluorescence intensity of HAS‐2 in PTC‐MP+NIR group was higher than that in PBS and Sham groups (Figure 8K,L), indicating that PTC‐MP hydrogel effectively protected HAS‐2 protein in synovial tissue.

## 3 Discussion and Prospects

**Figure 9:**
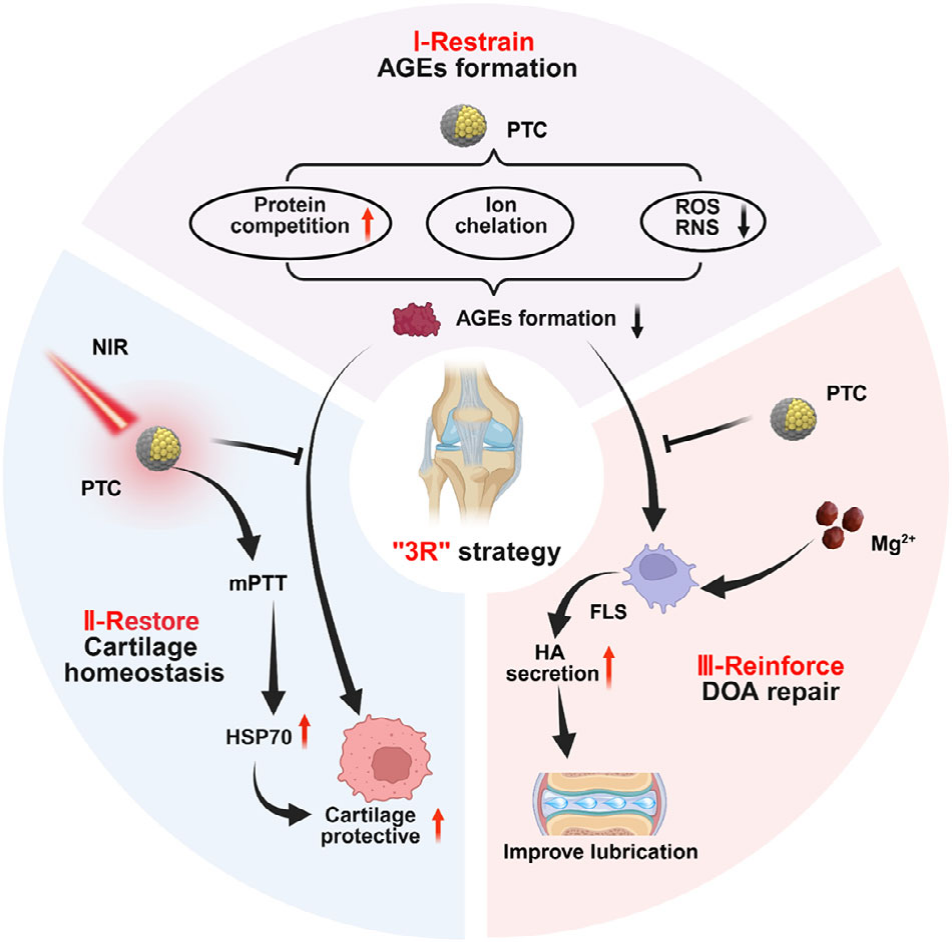
Mechanism of PTC‐MP exhibiting mPTT effects for AGEs‐directed DOA therapy. I‐PTC‐MP restrains AGEs formation; II‐PTC‐MP with mPTT restores cartilage homeostasis; III‐PTC‐MP reinforces DOA repair (Created with BioRender.com).

In this study, PTC‐MP hydrogel was successfully constructed based on the characteristic microenvironment of AGEs accumulation in DOA, and a “3R” (restrain‐restore‐reinforce) strategy was proposed to effectively treat DOA through a three‐pronged approach. To our knowledge, this is the first study to treat DOA based on AGEs target. Compared with previous studies, the advantages and limitations of this study are as follows:

(1) Although the pathological mechanism^[^ ^64^ ^]^ and treatment of OA^[^ ^65^ ^]^ have made remarkable progress, the research on the treatment of DOA, a special type of OA, is still lacking. AGEs are the key pathogenic factors of diabetic complications, and the accumulation of AGEs in diabetic patients is significantly higher than that in non‐diabetic patients, which accelerates the progression of DOA.^[^ ^66^ ^]^ Our research based on AGEs provides a novel approach for DOA treatment.

(2) Our “3R” strategy has improved the therapeutic effect of DOA from multiple perspectives. In vivo and in vitro experiments proved that PTC‐MP hydrogel restrained AGEs formation, restored cartilage homeostasis, and reinforced DOA repair by stimulating endogenous HA secretion to improve cartilage lubrication (**Figure** **9**).

However, our study has some limitations. For example, the animal model in this study was type I diabetes, whereas diabetic osteoarthritis (DOA) is more common in elderly patients with type II diabetes. The long‐term biodistribution, biodegradation, and ion releasing potential of nanomaterials in the joint cavity remain unclear.

## 4 Conclusion

A nanozyme‐integrated hydrogel PTC‐MP targeting AGEs is successfully constructed. PTC‐MP hydrogel with mPTT has a significant therapeutic effect on DOA model by inhibiting the generation of AGEs, up‐regulating the expression of HSP70, inhibiting AGEs‐induced inflammation and promoting HA secretion, and significantly improves motor function of rats. In summary, our “3R” treatment strategy targeting AGEs accumulation has opened up a novel approach for DOA therapy.

## 5 Experimental Section

### Materials

Chemical reagent: Bovine Serum albumin (BSA) and 8‐aniline‐1‐naphthalene sulfonate ammonium salt (ANS‐NH_4_) were purchased from Maclyn Biochemical Technology Co., Ltd. (Shanghai, China). Dopamine hydrochloride (DA‐HCl), trimethylol aminomethane hydrochloride (Tris‐HCl) buffer (pH 9.5), tannic acid (TA), cerium nitrate (Ce(NO_3_)_4_), sodium alginate (SA) and methylglyoxal (40%; MGO) were purchased from Aladdin Biochemical Technology Co., Ltd. (Shanghai, China). Coumarin 6 (Cou‐6), Sodium citrate buffer (pH 4.5) and Poloxam 407 (P407) were purchased from Solabio Life Sciences Co., Ltd. (Beijing, China). 1,1'‐dioctyl‐3,3,3',3'‐tetramethylindolecyl anthocyanine iodide (DiR) was purchased from Yeasen Biotechnology Co., Ltd. (Shanghai, China). Advanced glycation end products (AGEs) were purchased from Bioss Chemical. (Beijing, China). Streptozotocin (STZ) was purchased from Yuanye Bio‐Technology Co., Ltd. (Shanghai, China).

Biochemical reagents: Ferrous ion (Fe^2+^) content detection kit, magnesium test kit and superoxide dismutase (SOD) activity detection kit were purchased from Solabio Life Sciences Co., Ltd. (Beijing, China). Peptide sulfate hydrogen peroxide detection kit was purchased from Leagene Biotechnology Co., LTD. (Beijing, China). Mito‐Tracker Red‐CMXRos, Mitochondrial membrane potential assay kit with JC‐1 and Reactive oxygen species detection kit (DCFH‐DA) were purchased from Beyotime Biotechnology (Beijing, China). Ultra‐sensitive chemiluminescence detection kit (ECL), Dual color prestain protein Markers and BCA protein quantitative kit were purchased from Epizyme Biomedical Technology Co., Ltd. (Shanghai, China). Cell counting kit 8 (CCK‐8) was purchased from Biosharp Biotechnology Co., Ltd. (Beijing, China). Enzyme‐linked immunosorbent assay kits (ELISA) for tumor necrosis factor‐alpha (TNF‐α; Cat#JL13202), interleukin‐6 (IL‐6; Cat#JL20896), and matrix metalloproteinase‐13 (MMP‐13; Cat#JL10312) were purchased from Jianglai Biotechnology Co., Ltd. (Shanghai, China). Primary antibodies against B‐cell lymphoma 2 (BCL‐2; Cat#250412; 1:1000), BCl2‐associated X protein (BAX; Cat#380709; 1:1000), Cleaved‐caspase 3 (Cat#R23727; 1:1000), Nuclear Factor Kappa‐B (P65; Cat#R25150; 1:1000), Inhibitor of NF‐kappa‐B alpha (IKB‐α), Phosphorylated Nuclear Factor Kappa‐B (p‐P65; Cat#310013; 1:1000), Phosphorylated Inhibitor of NF‐kappa‐B alpha (p‐IKB‐α; Cat#340776;1:1000), receptor of advanced glycation end product (RAGE; Cat#R381618; 1:1000), heat shock protein 70 (HSP 70; Cat#R24633; 1:1000) and were purchased from ZEN‐BIOSCIENCE Co., Ltd. (Chengdu, China). Primary antibodies against aggrecan (ACAN; Cat#ab315486; 1:1000) and collagen‐II (COL‐II; Cat#ab307674; 1:1000) were purchased from Abcam plc (UK). Primary antibodies against hyaluronan synthase‐2 (HAS‐2; Cat#A9897; 1:1000) was purchased from ABclonal. (China). β‐Actin Mouse mAb (Cat#3010ES10) was purchased from Yeasen Biotechnology (Shanghai) Co., Ltd. Protease inhibitor cocktail and was purchased from Shandong Sparkjade Biotechnology Co., Ltd. NCM Western BlotStripping Buffer was purchased from New Cell & Molecular Biotech (China) Co., Ltd.

Cells and Animals: Chondrocytes and fibroblast‐like synovial cells (FLS) were purchased from Pricella Biotechnology Co., Ltd. (Wuhan, China). SD rats (Certification No. 370726240100668364) were purchased from Shandong Pengyue Laboratory Animal Science and Technology Co., Ltd. All animal experiments were approved by the Animal Protection and Use Committee of Ocean University of China (OUC‐SMP‐2024‐02‐21).

### Synthesis and Characterization of PTC Nanozymes

TA solution (24 mmol L^−1^) and Ce(NO_3_)_4_ solution (24 mmol/L) were mixed under alkaline conditions (pH 8.0, adjusted with NaOH), and stirred at room temperature for 5 h to synthesize tannic acid‐cerium (TA‐Ce). Then, DA‐HCl was introduced into the TA‐Ce solution with 20 mL of Tris‐HCl buffer (pH 9.5), followed by constant stirring (magnetic stirring, 1000 r min^−1^) at 40 °C for 24 h. After 2 days of dialysis (with a molecular weight cut‐off of 8 kDa) in deionized water, dopamine‐coating TA‐Ce was obtained and named as PTC. Detailed characterizations and bioactivity evaluation of PTC are provided in the Supporting Information.

### Preparation and Characterization of PTC‐MP

Sodium alginate (SA) (0.5%, w/v) was premixed with MgCl_2_ (0.1%, w/v), followed by addition of P407 (20%, w/v) under ice‐bath conditions to construct an interpenetrating network (IPN) hydrogel. PTC was subsequently incorporated to yield the composite hydrogel (PTC‐MP). The composite hydrogel of PTC and P407‐SA hydrogel (PTC‐P) and the composite hydrogel of MgCl_2_ and P407‐SA hydrogel (MP) were constructed by similar method. Detailed characterizations and bioactivity evaluation of PTC‐MP hydrogel are provided in the Supporting Information.

### Statistical Analysis

All results were presented as mean ± standard deviation (SD) and were repeated at least in triplicate. Data were plotted using GraphPad Prism 9.5. Statistical analysis was performed using IBM SPSS statistics 26. The differences between two groups were evaluated using a Student's *t*‐test. The differences among multi‐groups were performed using one‐way analysis of variance (ANOVA) with Tukey's multiple comparisons. Differences were classified statistically significant at a level: *p < 0.05, **p < 0.01, ***p < 0.001, ns means no statistical significance.

## Conflict of Interest

The authors declare no conflict of interest.

## Author Contributions

R.C. performed methodology, conceptualization, data curation, wrote original draft, preparation; Y.S., G.Z., and J.T. performed methodology, software; Q.D. and G.X. performed software, formal analysis; T.J. performed visualization; L.H. and X.Z. performed project administration, wrote, reviewed, and edited draft, and received funding acquisition, and performed supervision.

## Supporting information



Supporting Information

## Data Availability

The data that support the findings of this study are available from the corresponding author upon reasonable request.
