# Supplementary material for: Nanozyme‐Integrated Hydrogel Targeting AGEs for Diabetic Osteoarthritis Therapy
Source: Adv Sci (Weinh). 2025 Dec 5;13(8):e16389. doi: 10.1002/advs.202516389 (PMC12884749; doi:10.1002/advs.202516389)
Supplement: Supplementary file 1 — Supporting Information [file ADVS-13-e16389-s001.docx]

**Supporting Information**

**Nanozyme-Integrated Hydrogel Targeting AGEs for Diabetic Osteoarthritis Therapy**

Rui Chen ^a^, Yanguo Su ^a^, Guiyuan Zhao ^a^, Jiaojiao Tao ^a^, Qijie Diao ^a^, Guangli Xiang ^a^, Tianze Jiang ^a^, Lu Han ^a, b,*^, Xia Zhao ^a, b,^^[[1]](#footnote-1)^*

^a^ Key Laboratory of Marine Drugs, Ministry of Education, Shandong Key Laboratory of Glycoscience and Glycotherapeutics, School of Medicine and Pharmacy, Ocean University of China, Qingdao 266003, China.

^b^ Laboratory for Marine Drugs and Bioproducts, Qingda o Marine Science and Technology Center, Qingdao 266237, China.

Experimental Section

**1. Characterization of PTC**

The morphologies of TA-Ce and PTC were observed using transmission electron microscopy (TEM). The hydrated particle size distribution, polydispersion index (PDI) values and zeta potential of nanozymes were detected by dynamic light scattering detector (DLS) (Malvern Panalytical, UK). The cerium (Ce) ion concentration in PTC solution was determined by Agilent inductively coupled plasma mass spectrometer (ICP-MS). The structure of PTC was characterized by Fourier transform infrared spectroscopy (FT-IR) and Raman spectroscopy. The cerium dioxide (CeO_2_) content in PTC was determined by thermogravimetric analyzer (TGA) (Hitachi-STA200) provided by eceshi (www. eceshi.com). The crystal structure and valence distribution of PTC were characterized by X-ray diffraction (XRD) (Bruker-D8 ADVANCE) and X-ray photoelectron spectroscopy (XPS) (Thermo Fisher-K-Alpha), respectively.

The photothermal properties of PTC were investigated. PTC solution (1 mL) with different Ce concentrations (25, 50, 100 and 200 μg/mL) was added to quartz cuvettes. Samples were exposed to near-infrared (NIR) irradiation at different power (0.5, 1.0 and 1.5 W/cm²) for 10 min. Real-time temperature change of the samples was monitored using an infrared thermal imager. After each irradiation phase, samples were cooled to ambient temperature, followed by immediate initiation of subsequent heating-cooling cycles. The temperature curve of the samples was recorded at the same time.

**2. Characterization of PTC-MP hydrogel**

The surface morphology of PTC-MP hydrogel was observed by scanning electron microscopy (JEOL). The rheological properties of PTC-MP hydrogel were analyzed using rheometer (MCR302e, Anton Paar). The photothermal performance of PTC-MP hydrogel was measured by infrared image camera (FLIR E50, Estonia) at different Ce concentrations (25, 50, 100 and 200 μg/mL) under NIR (808 nm, 1 W/cm², 10 min) at different laser power densities (0.5, 1.0 and 1.5 W/cm²). To evaluate the photothermal stability, PTC-MP hydrogel (Ce concentration of 100 μg/mL) was irradiated under NIR (808 nm, 1 W/cm², 10 min) for 10 min. After each irradiation cycle, the laser was turned off to allow the sample to cool naturally for 10 min, and 7 cycles were repeated. Temperature change was recorded by infrared image camera. Photothermal conversion efficiency (η) was calculated using the following equations^[1]^.

η=[hS × (T_max_-T_sur_) - Q_Dis_)/(*I* × (1-10^-A808^)] × 100%

Where h indicates heat transfer coefficient; S indicates surface area of the container; T_max_ indicates equilibrium temperature; T_sur_ indicates ambient temperature of the surrounding; Q_Dis_ is the absorbed energy by water; *I* represents laser power density; A808 is absorbance of sample at 808 nm.

**3. Effect and mechanism of PTC inhibiting the formation of AGEs**

An experimental model of pyruvate-bovine serum protein (MGO-BSA) glycation system was constructed by mixing BSA (1.0 mg/mL), MGO (5.0 mmol/L) and PTC with different Ce concentrations (50, 100 and 200 μmol/L) under oscillated in a 37°C water bath for 7 days^[2]^. The fluorescence intensity of AGEs was quantified using fluorescence enzyme spectrometer (excitation wavelength at 370 nm, emission wavelength ranging from 410 to 460 nm), and the inhibition rate of AGEs was calculated using the following formula: Inhibition rate (%) = [(F _control_ - F _sample_) / F _control_] × 100%. F _control_ ​ and F _sample_ ​represent the fluorescence intensities of the PBS treated sample and PTC treated sample, respectively.

The glycosylated system was incubated with the hydrophobic fluorescence probe ANS-NH_4_ (5 mmol/L) for 5 min. Fluorescence spectrum changes were recorded using fluorescence spectrometer (excitation wavelength at 380 nm, emission wavelength ranging from 430 to 600 nm). The inhibition rate of BSA's hydrophobic structure were calculated using the following formula: Inhibition rate (%) = [(F_control_ - F_sample_) / F_control_] × 100%. F_control_ ​and F_sample_ ​represent the fluorescence intensities of the PBS treated sample and PTC treated sample, respectively.

PTC with different Ce concentrations (50, 100 and 200 μmol/L) were added to FeCl_2_ solution (400 μmol/L) at 37 ℃ for 3 h. Residual Fe^2+^ content was quantified by Fe^2+^ content detection kit. Absorbance changes within wavelength range of 400–700 nm were measured via ultraviolet-visible (UV-Vis) spectroscopy. The Fe²⁺ chelation rate was calculated using the following equation: Chelation rate (%) = [(OD_control_ - OD _sample_)/OD_control_] × 100%. OD_control_ and OD_sample_ represent the absorbance values of the PBS treated sample and PTC treated sample, respectively.

**4. ROS and RNS scavenging ability of PTC**

The superoxide anion radical (O^2-^) scavenging ability of PTC at different Ce concentrations (50, 100 and 200 μg/mL) under NIR irradiation was evaluated using the SOD detection kit. The hydrogen peroxide (H_2_O_2_) scavenging ability of PTC at different Ce concentrations (50, 100 and 200 μg/mL) under NIR irradiation was evaluated using peptide sulfate hydrogen peroxide detection kit. The SOD and CAT activities of PTC nanozyme between day 1 and day 10 under NIR or non-NIR conditions were further evaluated. Dissolved oxygen production was detected using a dissolved oxygen meter, and the formation of O_2_ bubbles was photographed. The hydroxyl radical (·OH) scavenging ability of PTC was detected by 3,3',5,5' -tetramethylbenzidine (TMB) method. Briefly, H_2_O_2_ (5 mmol/L) was pre-mixed with Fe^2+^ (2 mmol/L) to generate ·OH, followed by different concentrations of PTC. After full reaction, TMB solution was added to supernatant, and the absorbance change within the 500-1000 nm range was recorded via UV-visible spectrophotometer. TMB was used as the substrate to detect ROS produced by PTC under NIR.

2, 2-diphenyl-1-pyridinium (DPPH·) (0.4 mmol /L) was mixed with PTC solutions, and the absorbance change within the 450-700 nm range was recorded via UV-visible spectrophotometer. Total antioxidant capacity of PTC was evaluated using the Total antioxidant capacity assay kit (2,2 '-diazo-bis -(3-ethylbenzothiazolin-6-sulfonic acid) (ABTS^+·^) method) (Beyotime, S0119), with absorbance measured at 450-700 nm using UV-visible spectrophotometer.

**5.** **PTC and Mg^2+^ biocompatibility test**

Chondrocytes and FLS (2×10^4^ cells per well) were seeded into a 96-well plate and cultured for 12 h. Then, different concentrations of PTC and Mg^2+^ were added and continued for 24 h, respectively. The cell viability was tested by CCK-8 kit instructions.

Hemolysis testing was performed by incubating fresh rat erythrocytes at different Ce concentrations (0, 50, 100 and 200 μmol/L) of PTC-MP hydrogel in PBS for 6 h. Control group included PBS (negative control) and 1% Triton X-100 (positive control). The UV absorption value of the supernatant at 540 nm was determined.

**6. *In vitro* Mg^2+^ release**

The dialysis membrane (with a molecular weight cut-off of 3.5 kDa) with PTC-MP hydrogel (1 mL) were put into 30 mL PBS release medium under shaking in a water bath at 37 ℃. The release medium was removed and then replaced by the same volume of fresh PBS at set intervals. To evaluate the effect of NIR on the *in vitro* release of Mg^2+^, the PTC-MP hydrogel was irradiated with NIR (808 nm, 1 W/cm^2^) or without NIR. The concentration of Mg^2+^ was measured by a magnesium test kit.

**7. *In vitro* cell internalization of PTC under mPTT**

Cou-6 (a fluorescent dye) was employed to label PTC for the construction of PTC-MP@Cou-6 hydrogel. Chondrocytes and FLS (2×10^5^ cells per well) were seeded into a 6-well plate for 24 h. Then, the media was replaced by a fresh culture medium with PTC-MP@Cou-6. After 30 min of incubation, the cells were exposed to NIR irradiation (808 nm, 1 W/cm^2^, 10 min) and designated as PTC-MP@Cou-6+NIR. The non-irradiation group was named as PTC-MP@Cou-6. Then, the cells were fixed with 4% paraformaldehyde (PFA) and stained with Hoechst 33342. Finally, cell internalization of PTC in PTC-MP hydrogel was evaluated by laser scanning confocal microscopy (CLSM) (Leica, Germany) and flow cytometry (FCM) (Beckman, USA), respectively.

**8. Immunofluorescent staining**

Chondrocytes and FLS (2×10^5^ cells per well) were seeded into a 6-well plate and cultured for 24 h. Cells were divided into 5 groups: (1) PTC-MP hydrogel under NIR irradiation (808 nm, 1 W/cm², 10 min) (PTC-MP+NIR), (2) PTC-MP hydrogel, (3) MP hydrogel under NIR irradiation (808 nm, 1 W/cm², 10 min) (MP+NIR), (4) PTC-P hydrogel under NIR irradiation (808 nm, 1 W/cm², 10 min) (PTC-P+NIR), (5) PBS treatment. After 4 h, the medium was removed and AGEs (100 μg/mL) were added for 24 h of stimulation. The Sham group was untreated with AGEs. Then treated cells were fixed with 4% paraformaldehyde and treated with 0.5% Triton X-100. After being blocked with BSA for 30 min, chondrocytes and FLS were incubated with primary antibodies against cartilage marker proteins (ACAN, COL-2) and HAS-2, respectively. Then, alexa fluor 488 (AF488)-labeled secondary antibodies (1:200) were incubated for 1 h at room temperature in the dark. Finally, cell nuclei were stained with Hoechst 33342 and observed using CLSM.

**9. Intracellular ROS level and Inflammatory cytokine expression**

Chondrocytes and FLS (2×10^5^ cells per well) were seeded in a 6-well plate for 12 h. Then, the cells were treated with different groups (PTC-MP+NIR; PTC-MP; MP+NIR; PTC-P+NIR; PBS; Sham). Commercial sodium hyaluronate (HA) gel, as a horizontal contrast, was compared with PTC-MP hydrogel for ROS scavenging. After 30 min incubation with DCFH-DA, ROS levels were analyzed using CLSM and FCM, respectively. The expression levels of inflammatory cytokines (IL-6, MMP-13 and TNF-α) in the supernatant of Chondrocytes and FLS in different groups (PTC-MP+NIR; PTC-MP; MP+NIR; PTC+NIR; PBS; Sham) were quantified by ELISA, respectively.

**10. Mitochondrial function test in chondrocytes**

Chondrocytes (2×10^5^ cells per well) were seeded in 6-well plates for 12 h. Then, the cells were treated with different groups (PTC-MP+NIR; PTC-MP; MP+NIR; PTC+NIR; PBS; Sham). Then, the cells were incubated with JC-1 working solution (5 μg/mL) and Mito-Tracker Red CMXRos (100 nmol/L), respectively. Fluorescent images were observed by CLSM, and the staining results were analyzed by Image J software.

**11. Western blotting (WB) analysis in chondrocytes**

Chondrocytes (2×10^5^ cells per well) were seeded in 6-well plates for 12 h. Then, the cells were treated with different groups (PTC-MP+NIR; PTC-MP; MP+NIR; PTC+NIR; PBS; Sham). The samples were separated using 4-20% sodium dodecyl sulfate-polyacrylamide gel electrophoresis (SDS-PAGE) at 150 V for 40 min after protein extraction from chondrocytes. Subsequently, the proteins were transferred to PVDF membranes for immobilization. The membranes were incubated overnight at 4°C with primary antibodies against RAGE, HSP-70, ACAN, COL-II, BCL-2, BAX, cleaved caspase-3, P65, p-P65, IKB, p-IKB and β-actin. Then, the membranes were incubated with secondary antibodies for 2 h at room temperature. The signals were detected using Enhanced Chemiluminescence Liquid (ECL) and quantified by Image J software.

**12. HA secretion and HAS-2 expression in FLS**

FLS (2×10^5^ cells per well) were seeded in glass bottom cell culture dish (Guangzhou Jet Bio-Filtration Co., Ltd.) for 12 h. Then, the cells were treated with different groups (PTC-MP+NIR; PTC-MP; MP+NIR; PTC+NIR; PBS; Sham). WB assay was performed to analyze HAS-2 expression. The expression of hyaluronic acid (HA) in different groups was detected by ELISA.

**13. *In situ* mPTT effect and retention experiment of PTC-MP**

PTC-MP (50 μL) was administered via intra-articular injection to the joint cavity followed by NIR irradiation (808 nm) with different power (0.1, 0.2, 0.4 and 1 W/cm^2^). The temperature change in the knee joint was monitored by infrared image camera. MP (50 μL) served as the control formulation through identical intra-articular administration. DiR (1 mg/kg) was incorporated to construct fluorescent PTC-MP@DiR hydrogels. The labeled hydrogels (50 μL) were subsequently injected intra-articularly. The retention of hydrogel *in vivo* was investigated by Live animal imaging. The rats were euthanized and their major organs (heart, liver, spleen, lungs, kidneys) were removed for further imaging analysis on day 7.

**14. Construction of diabetic osteoarthritis (DOA) model in SD rats**

Male Sprague-Dawley (SD) rats (160-180 g) were obtained from Shandong Pengyue Laboratory Animal Science and Technology Co., Ltd. All animal experiments were approved by the Animal Protection and Use Committee of Ocean University of China (OUC-SMP-2024-02-21). To establish diabetic models, rats fasted for 12 h and received intraperitoneal injections of streptozotocin (STZ; 10 mg/mL) at 50 mg/kg daily for three consecutive days. Rats with fasting blood glucose levels above 16.5 mmol/L were selected for subsequent osteoarthritis (OA) modeling by anterior cruciate ligament transection (ACLT). Under anesthesia, the right hind limb of diabetic rats was sterilized with ethanol. A longitudinal incision (3 cm) was made medial to the knee joint, followed by dissection along the patellar ligament. The muscle was incised along the ligaments, and the knee was flexed and the patella was turned over. After cruciate ligament incision, a drawer test was performed to confirm the establishment of DOA model, and then the wound was sutured layer by layer. Diabetic SD rats without ACLT surgery were randomly selected as Sham group.

Animals were randomly divided into 6 experimental groups (n=6): (1) PTC-MP+NIR; (2) PTC-MP; (3) MP+NIR; (4) PTC-P+NIR; (5) PBS; (6) Sham. Weekly therapeutic interventions were administered according to group assignments, with concurrent monitoring of fasting blood glucose levels. NIR irradiation parameters were standardized at the wavelength of 808 nm, the power density of 0.4 W/cm², duration10 min and once a week. After 7 weeks, all rats were killed, and their joints were harvested and decalcified for subsequent staining experiments. H&E staining of major organs (heart, liver, spleen, lungs, kidneys) was performed to evaluate the biosafety of PTC-MP.

**15. Therapeutic effect of PTC-MP with mPTT against DOA *in vivo***

Micro-CT analysis and H&E staining were performed to evaluate cartilage degradation and joint space dimensions in rat knee joints in different groups. Alcian blue (AB) and safranin O-Fast Green (SO-FG) staining were evaluate collagen deposition levels and cartilage thickness, respectively. H&E staining and Masson staining were applied to evaluate synovial inflammatory infiltration. At the 5th and 7th week of the experiment, the rat paws were marked with red (left paw) and blue (right paw) dyes and then walked naturally on white paper, and the footprints were collected for statistical analysis.

Supplementary Figures

**
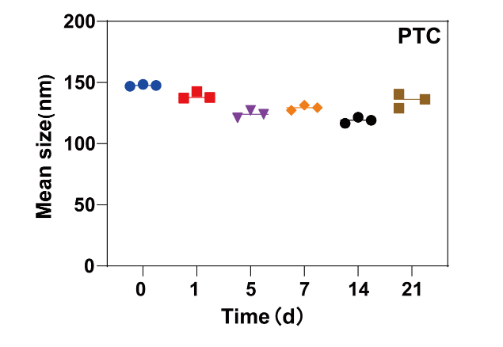
**

**Figure S1**. Size change of PTC over a period of 21 d. Results are presented as mean ± SD (n=3).


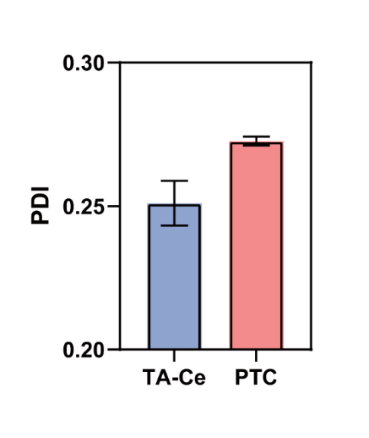


**Figure S2**. PDI values of TA-Ce and PTC. Results are presented as mean ± SD (n=3).


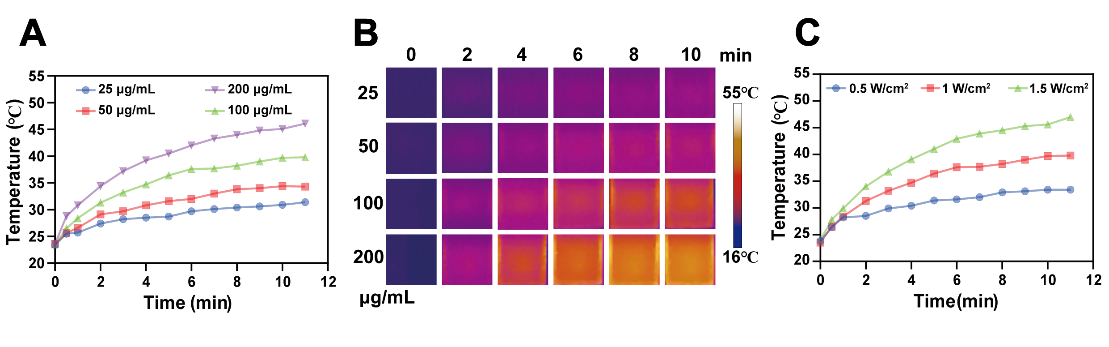


**Figure S3**. Mild photothermal effect of PTC. Temperature curves (A) and infrared thermal image (B) of PTC with different Ce concentrations (25, 50, 100 and 200 μg/mL) under NIR (808 nm, 1.0 W/cm^2^). (C) Temperature curves of PTC (Ce concentration of 100 μg/mL) under NIR with different powers (0.5, 1.0 and 1.5 W/cm^2^).

**
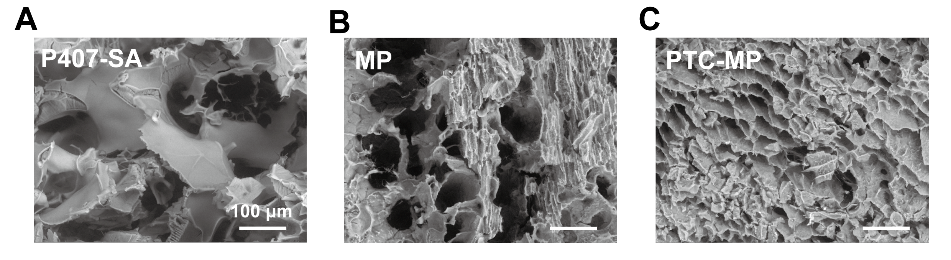
**

**Figure S4**. Characterization of PTC-MP. SEM images of P407-SA (A), MP (B), and PTC-MP (C).


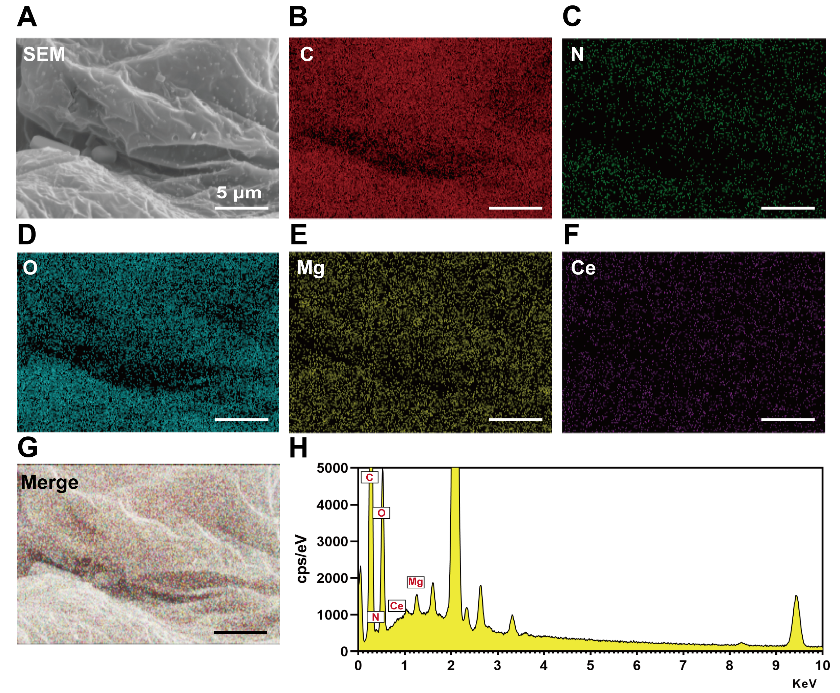


**Figure S5**. Elemental mapping images of PTC-MP hydrogel. (A) Topography of PTC-MP hydrogel. Elemental distribution characterization on the surface of PTC-MP hydrogel, C (B), N (C), O (D), Mg (E), Ce (F) and merge mapping (G). (H) Energy Dispersive X-ray Spectroscopy (EDS) analysis of PTC-MP hydrogel.


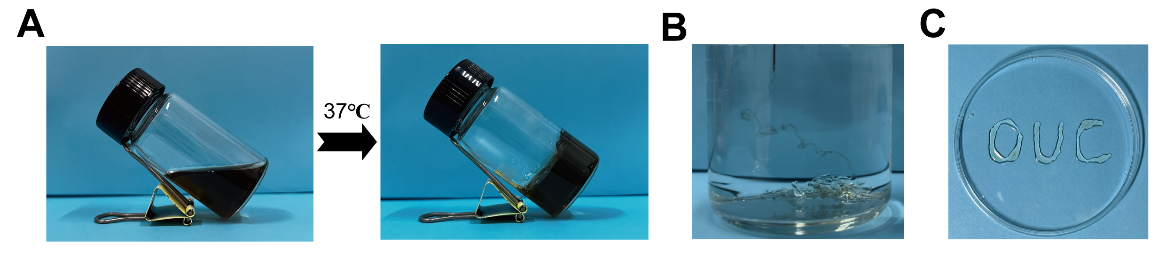


**Figure S6**. Gelation properties of PTC-MP hydrogel. (A) Sol-gel phase transition images of PTC-MP hydrogel at 37 ℃. (B) Injectability performance of PTC-MP hydrogel. (C) Rapid gelatinization performance of PTC-MP hydrogel at 37 ℃.


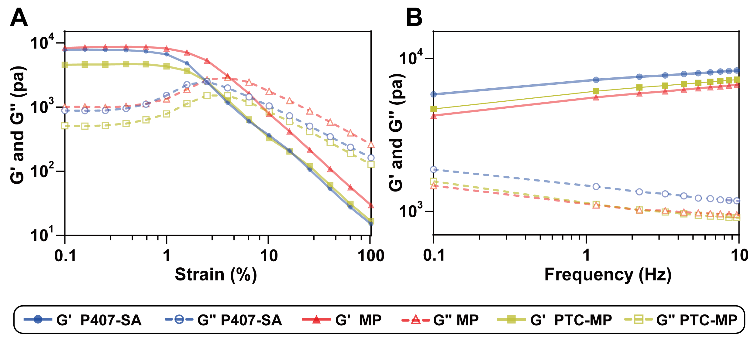


**Figure S7**. Rheological properties of PTC-MP hydrogel. Strain dependence (A) and frequency dependence (B) of storage modulus (G', solid symbols) and loss modulus (G'', open symbols) performance of P, MP and PTC-MP hydrogel.


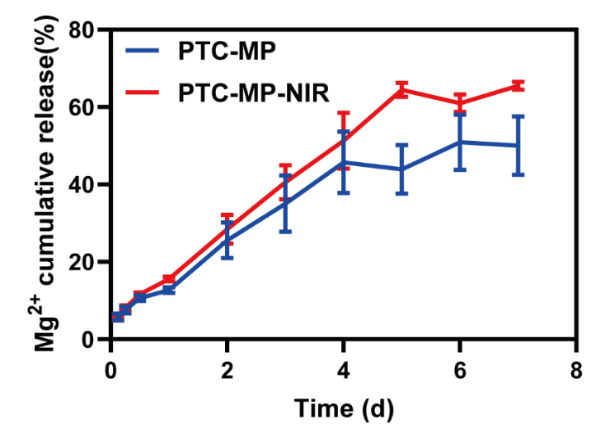


**Figure S8.** *In vitro* cumulative release curves of Mg^2+^ from PTC-MP under NIR or non-NIR condition (n=3). Data are presented as mean values ± SD.


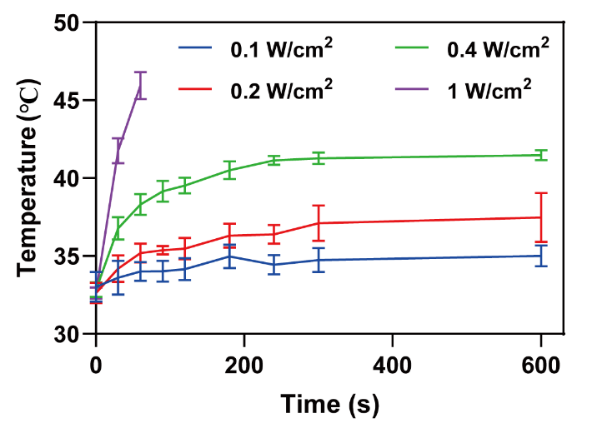


**Figure S9.** *In vivo* photothermal effect of PTC-MP hydrogel under NIR with different powers (0.1, 0.2, 0.4 and 1 W/cm^2^) (n=6). Data are presented as mean values ± SD.

**
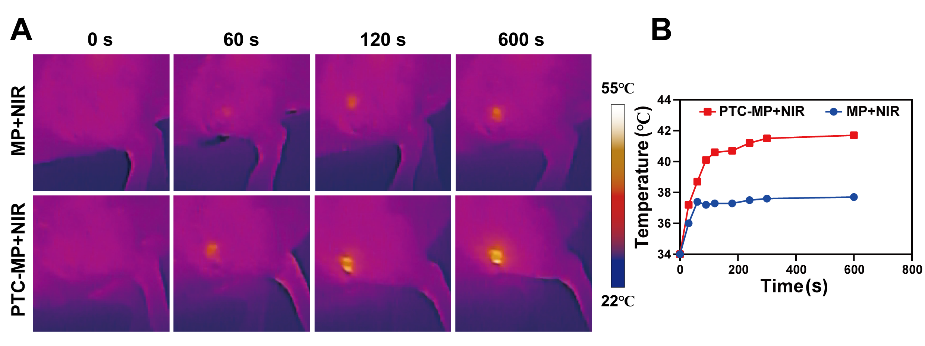
**

**Figure S10**. Mild photothermal effect of PTC-MP hydrogel *in vivo*. Infrared thermal image (A) and temperature curves (B) of PTC-MP hydrogel (Ce concentration of 400 μg/mL) and MP hydrogel under NIR (808 nm, 0.4 W/cm^2^).


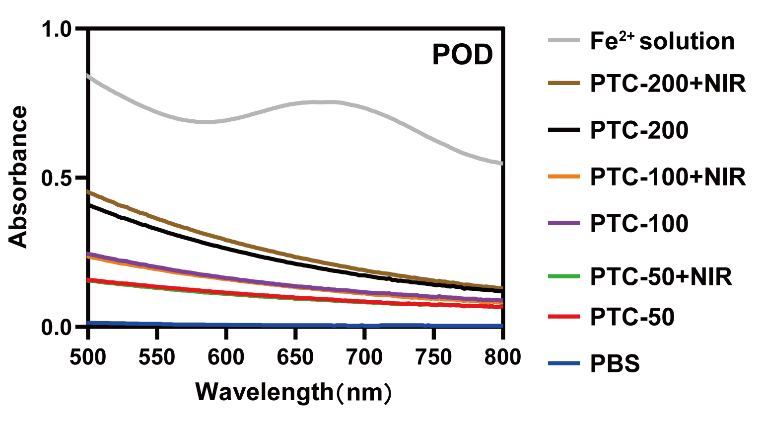


**Figure S11**. ROS production of PTC system under NIR detected by TMB as the probe.


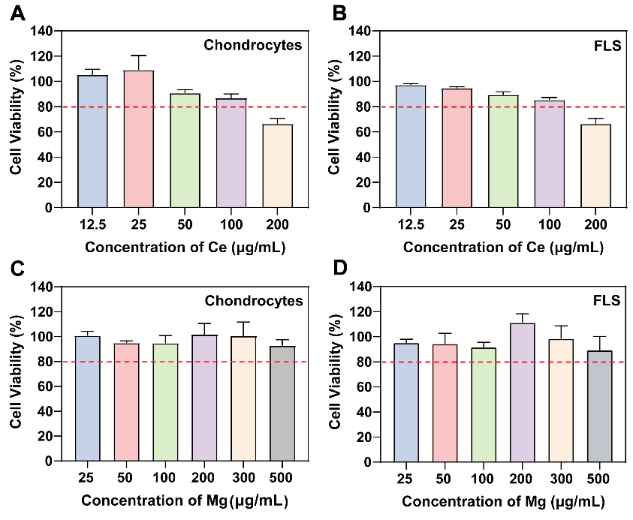


**Figure S12.** Cytotoxicity of PTC and Mg^2+^. Cell viability of chondrocytes (A) and FLS (B) treated with different concentrations of PTC for 24 h. Cell viability of chondrocytes (C) and FLS (D) treated with different concentration of Mg^2+^ for 24 h. All data were shown as mean ± SD (n=3).


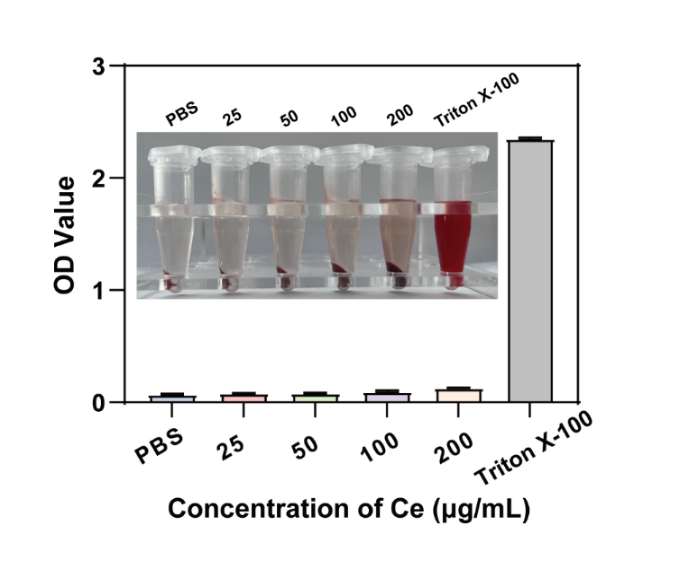


**Figure S13**. Hemolytic assay of PTC with different Ce concentrations (25, 50, 100, and 200 μg/mL) (n=4).


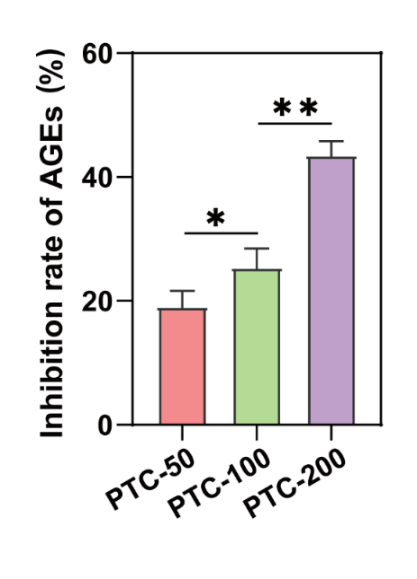


**Figure S14**. Inhibition rate of PTC on AGEs formation (n=6). Data are presented as mean values ± SD. Comparisons were performed by one-way ANOVA followed by Tukey’s multiple comparisons test. (**P < 0.05, **P < 0.01*).


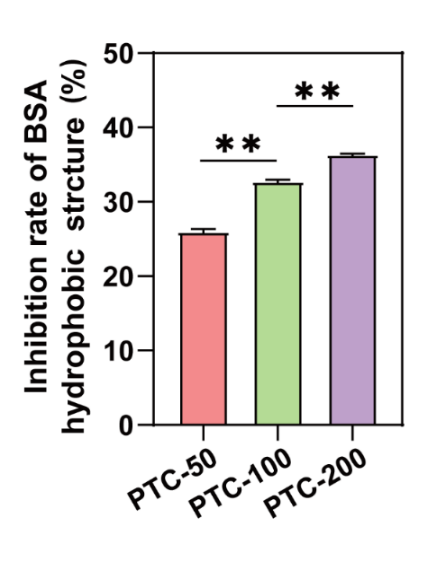


**Figure S15**. Inhibition rate of the hydrophobic structure of BSA (n=5). Data are presented as mean values ± SD. Comparisons were performed by one-way ANOVA followed by Tukey’s multiple comparisons test. (***P < 0.01*).


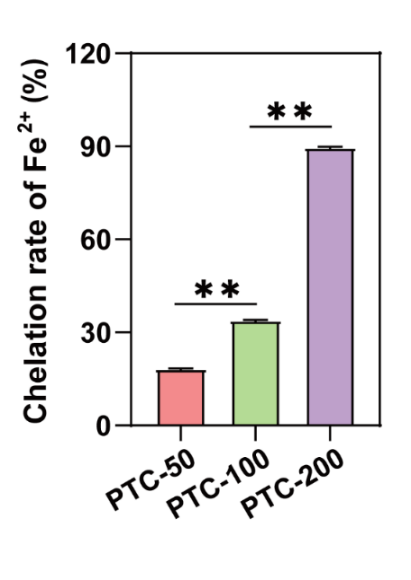


**Figure S16**. Chelation rate of PTC with Fe^2+^ (n=6). Data are presented as mean values ± SD. Comparisons were performed by one-way ANOVA followed by Tukey’s multiple comparisons test (***P < 0.01*).


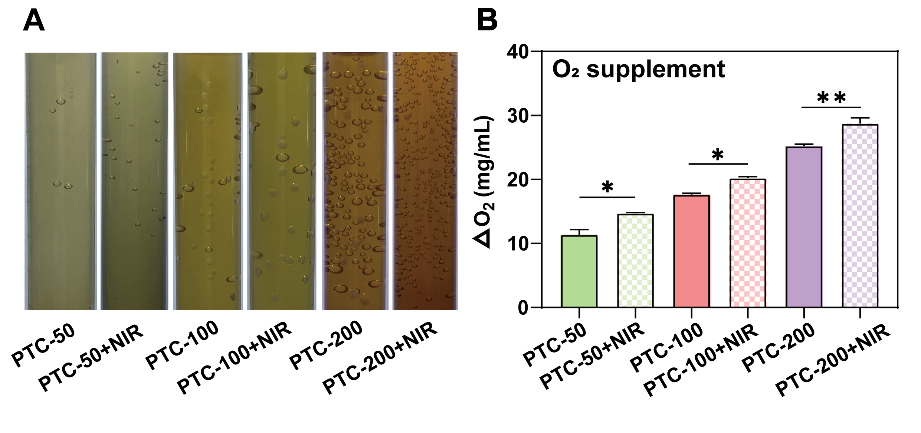


**Figure S17**. CAT activity of PTC under NIR or non-NIR. (A) Digital photographs of O_2_ production in H_2_O_2_ solutions by different concentrations of PTC. (B) Dissolved oxygen levels in H_2_O_2_ solutions by different concentrations of PTC (n=3). Data are presented as mean values ± SD. Comparisons were performed by one-way ANOVA followed by Tukey’s multiple comparisons test. (**P < 0.05, **P < 0.01*).


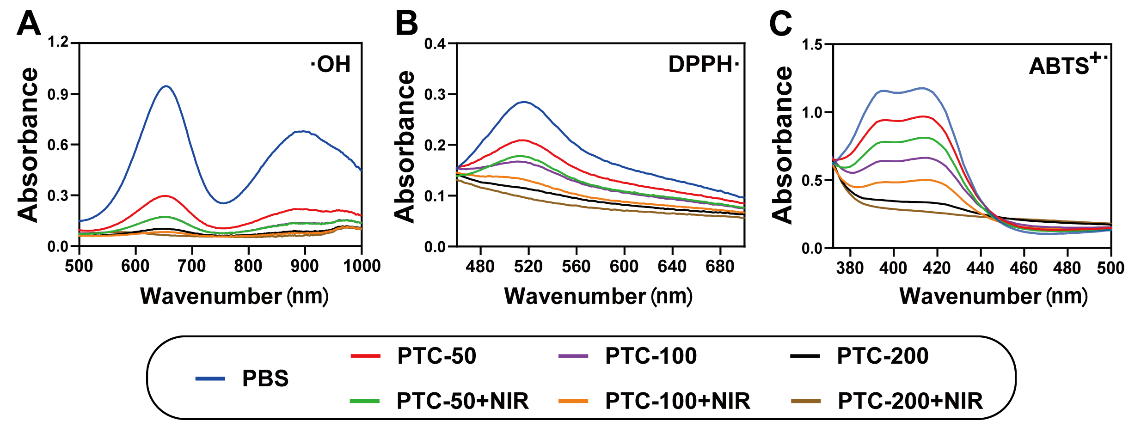


**Figure S18**. UV-vis absorbance spectra of antioxidases-mimicking capacities of PTC with different concentration under NIR or non-NIR for ·OH (A), DPPH· (B) and ABTS^+·^ (C).


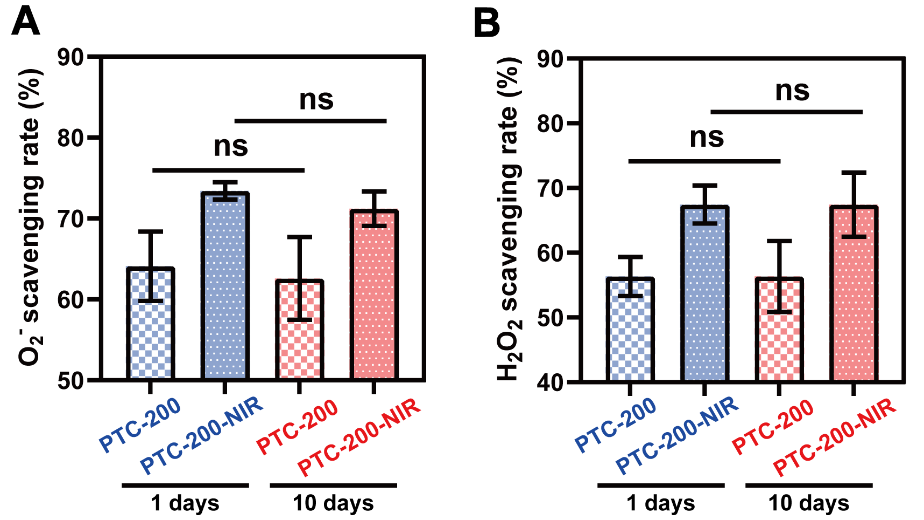


**Figure S19**. SOD-like and CAT-like activities long-term enzymatic stability of PTC. Quantitative analysis of O_2_^•−^ (A) and H_2_O_2_ (B) scavenging rates of PTC under NIR or non-NIR on day 1 and day 10 (n=5). Data are presented as mean values ± SD. Comparisons were performed by unpaired two-tailed Student’s t test (ns means no statistical significance).


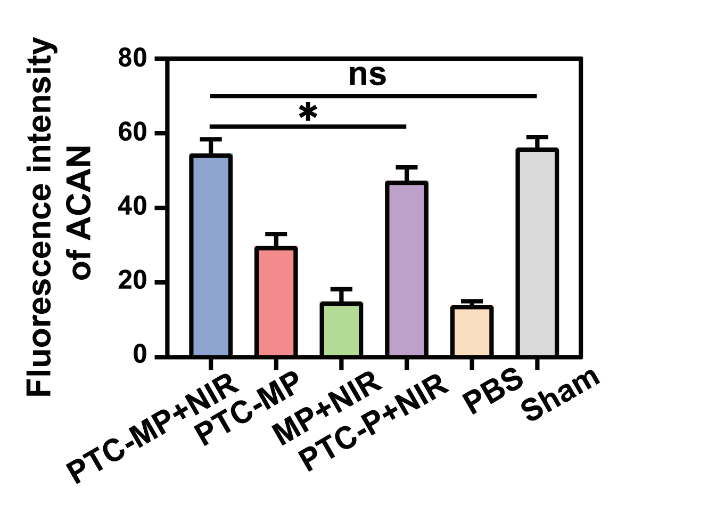


**Figure S20**. Semi-quantitative analysis of ACAN expression in chondrocytes treated with different groups (n=3). Data are presented as mean values ± SD. Comparisons were performed by one-way ANOVA followed by Tukey’s multiple comparisons test. (**P < 0.05,* ns means no statistical significance).


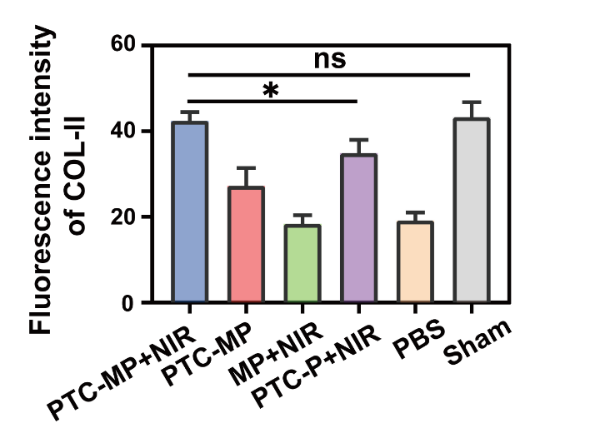


**Figure S21**. Semi-quantitative analysis of COL-II expression in chondrocytes treated with different groups (n=3). Data are presented as mean values ± SD. Comparisons were performed by one-way ANOVA followed by Tukey’s multiple comparisons test. (**P < 0.05,* ns means no statistical significance)


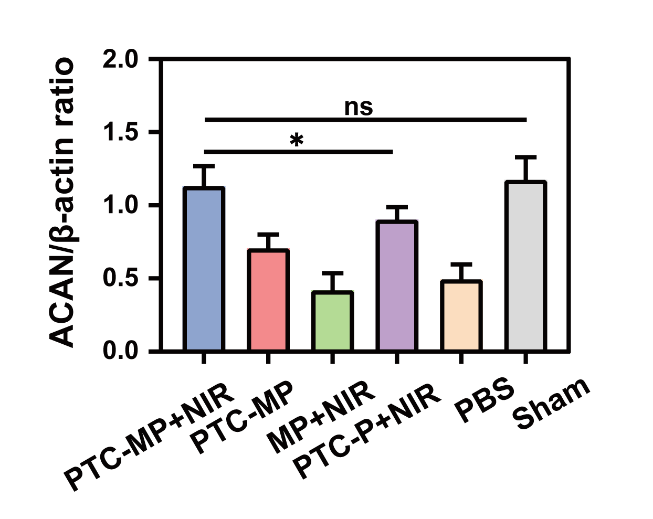


**Figure S22**. Quantitative analysis of ACAN expression in chondrocytes after treated with different groups (n=5). Data are presented as mean values ± SD. Comparisons were performed by one-way ANOVA followed by Tukey’s multiple comparisons test. (**P < 0.05,* ns means no statistical significance).


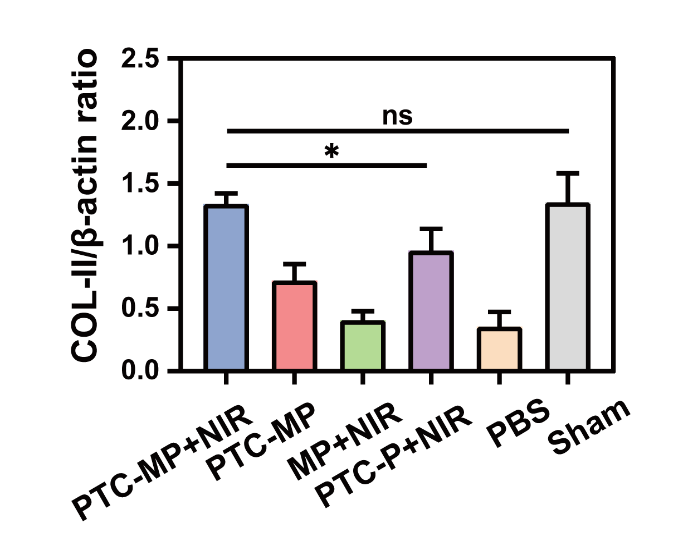


**Figure S23**. Quantitative analysis of COL-II expression in chondrocytes after treated with different groups (n=4). Data are presented as mean values ± SD. Comparisons were performed by one-way ANOVA followed by Tukey’s multiple comparisons test. (**P < 0.05,* ns means no statistical significance).


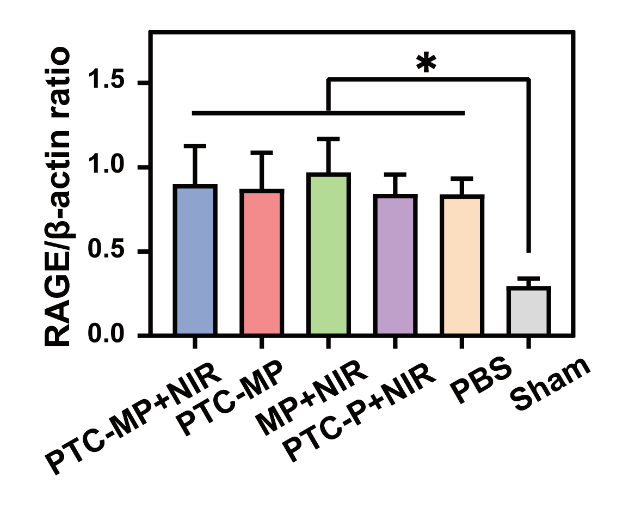


**Figure S24**. Quantitative analysis of RAGE expression (n=3). Data are presented as mean values ± SD. Comparisons were performed by one-way ANOVA followed by Tukey’s multiple comparisons test. (**P* < 0.05)


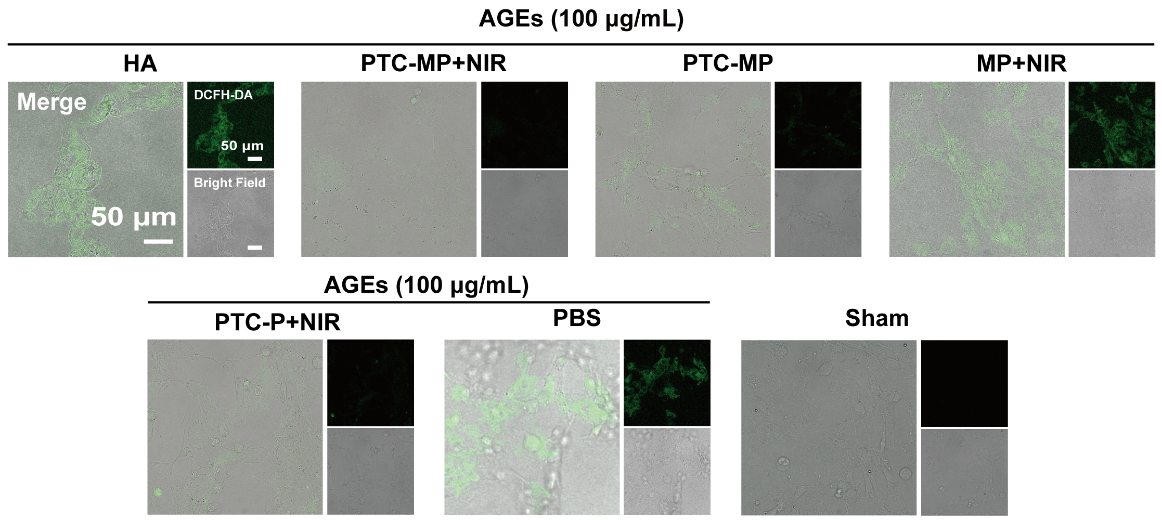


**Figure S25**. Fluorescence images of intracellular ROS levels in chondrocytes incubated with different groups: Chondrocytes were pre-treated with commercially available Sodium Hyaluronate gel as horizontal contrast group (HA), PTC-MP hydrogel under NIR irradiation (808 nm, 1 W/cm², 10 min, PTC-MP+NIR), PTC-MP hydrogel (PTC-MP), MP hydrogel under NIR irradiation (808 nm, 1 W/cm², 10 min, MP+NIR), PTC-P hydrogel under NIR irradiation (808 nm, 1 W/cm², 10 min, PTC-P+NIR), PBS treatment (PBS). The untreated group was regarded as Sham group.


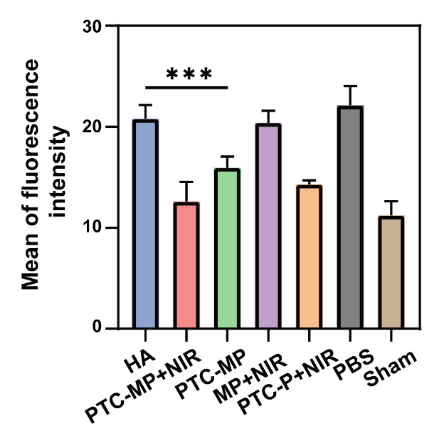


**Figure S26**. Semi-quantitative analysis of intracellular ROS levels in chondrocytes treated with different groups (n=5). Data are presented as mean values ± SD. Comparisons were performed by one-way ANOVA followed by Tukey’s multiple comparisons test. (***P < 0.001).


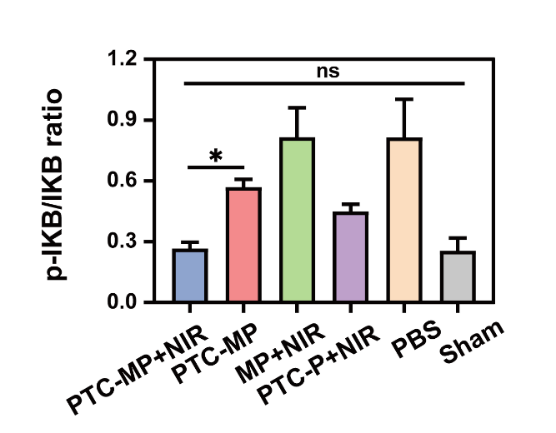


**Figure S27**. Quantitative analysis of p-IKB expression (n=4). Data are presented as mean values ± SD. Comparisons were performed by one-way ANOVA followed by Tukey’s multiple comparisons test. (**P < 0.05,* ns means no statistical significance)


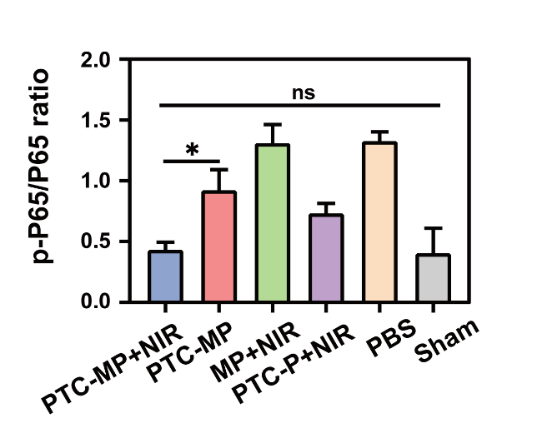


**Figure S28**. Quantitative analysis of p-P65 proteins expression. All data were shown as mean ± SD (n=4). Data are presented as mean values ± SD. Comparisons were performed by one-way ANOVA followed by Tukey’s multiple comparisons test. (**P < 0.05,* ns means no statistical significance)


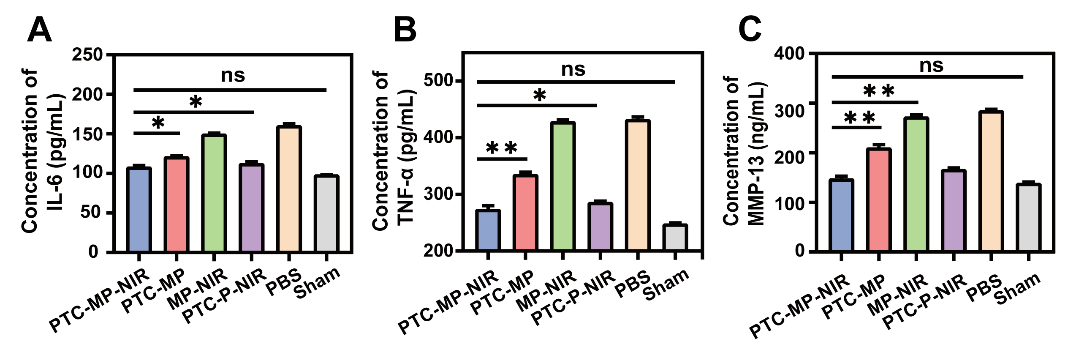


**Figure S29.** Expression of inflammatory cytokines levels in chondrocytes. ELISA assay quantification of IL-6 (A), TNF-α (B) and MMP-13 (C) expression in different groups (n=3). Data are presented as mean values ± SD. Comparisons were performed by one-way ANOVA followed by Tukey’s multiple comparisons test. (**P < 0.05, **P < 0.01,* ns means no statistical significance)


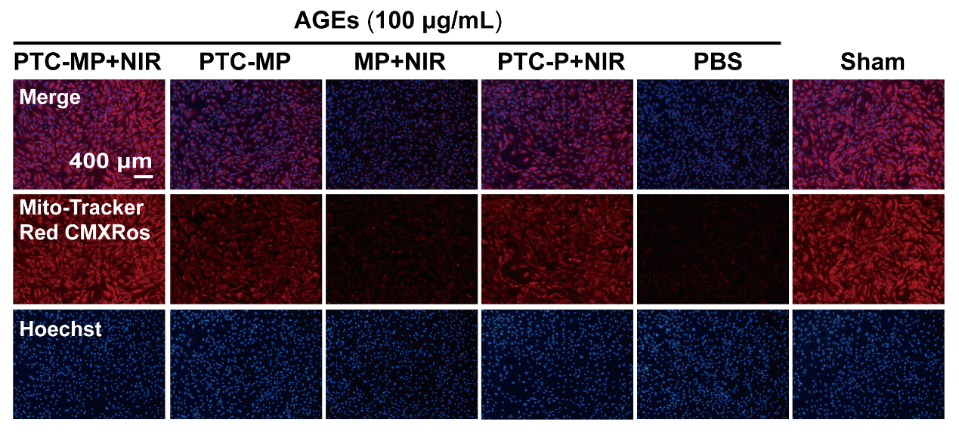


**Figure S30.** Fluorescence images of chondrocytes stained with Mito-Tracker Red CMXRos staining in different groups.


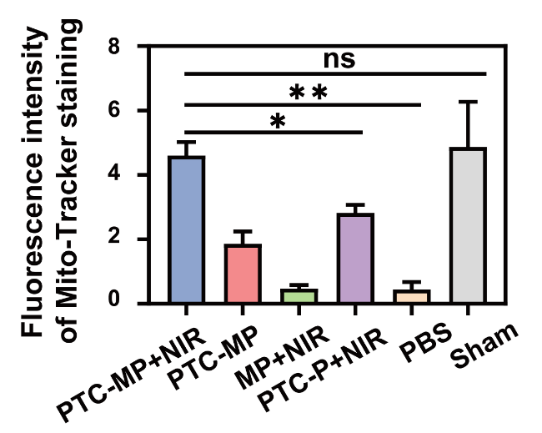


**Figure S31.** Semi-quantitative analysis of chondrocytes stained with Mito-Tracker Red CMXRos staining in different groups (n=3). Data are presented as mean values ± SD. Comparisons were performed by one-way ANOVA followed by Tukey’s multiple comparisons test. (**P < 0.05, **P < 0.01,* ns means no statistical significance)

**
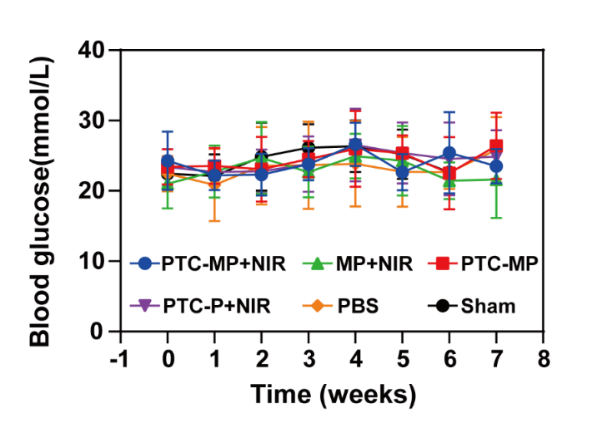
**

**Figure S32**. Changes of blood glucose in rats throughout the treatment (n=6).


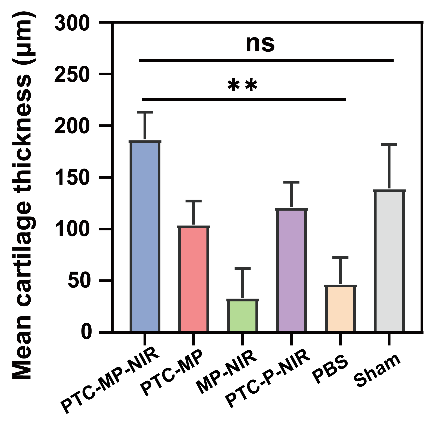


**Figure S33**. Mean cartilage thickness after different treatment in different groups (n=6). Data are presented as mean values ± SD. Comparisons were performed by one-way ANOVA followed by Tukey’s multiple comparisons test. (***P < 0.01,* ns means no statistical significance)


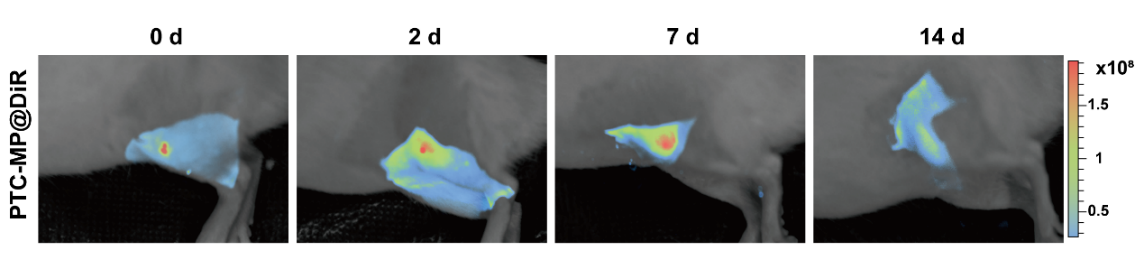


**Figure S34**. Fluorescence imaging of SD rats over 14 d after single intra-articular injection of PTC-MP@DiR *in situ*.


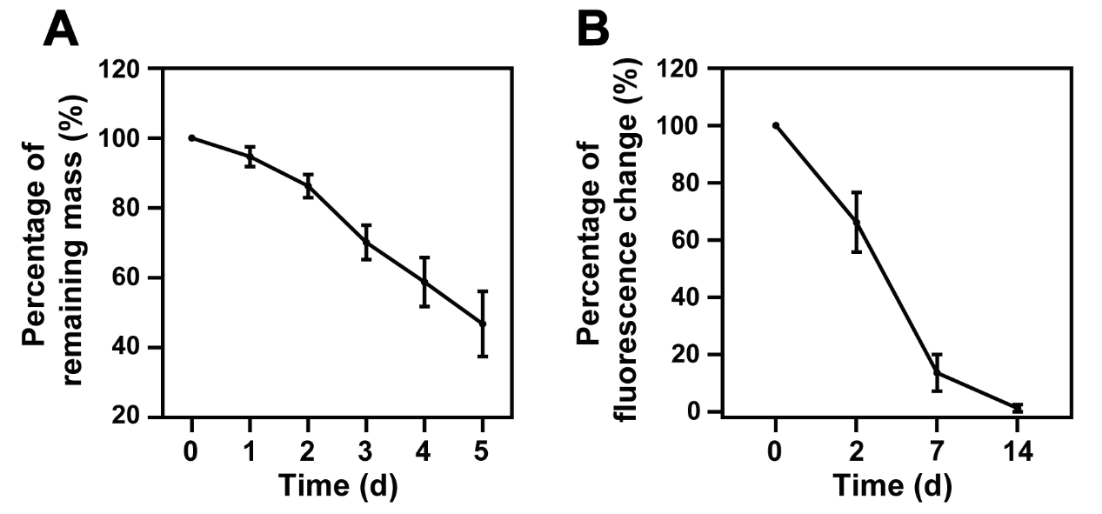


**Figure S35.** Percentage of remaining mass and flurescence change of PTC-MP hydrogel. (A) Percentage of remaining mass of PTC-MP hydrogel in the simulated synovial fluid (n=6). Data are presented as mean values ± SD. (B) Quantification analysis of flurescence change of PTC-MP hydrogel in the joint cavity *in situ* (n=5). Data are presented as mean values ± SD.


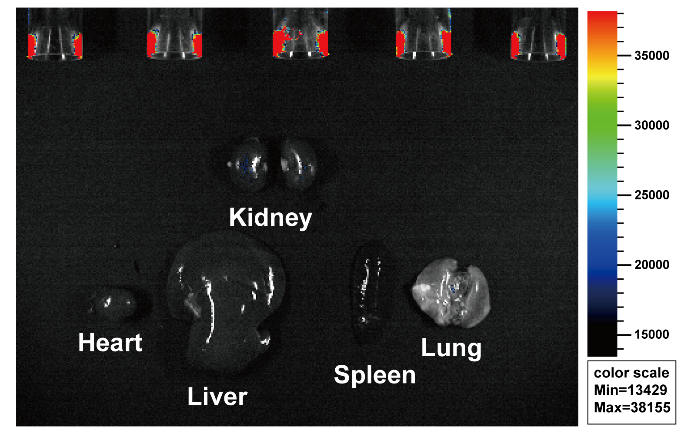


**Figure S36**. Fluorescence distribution in *ex vivo* heart, liver, spleen, lungs, kidneys on day 7.

**
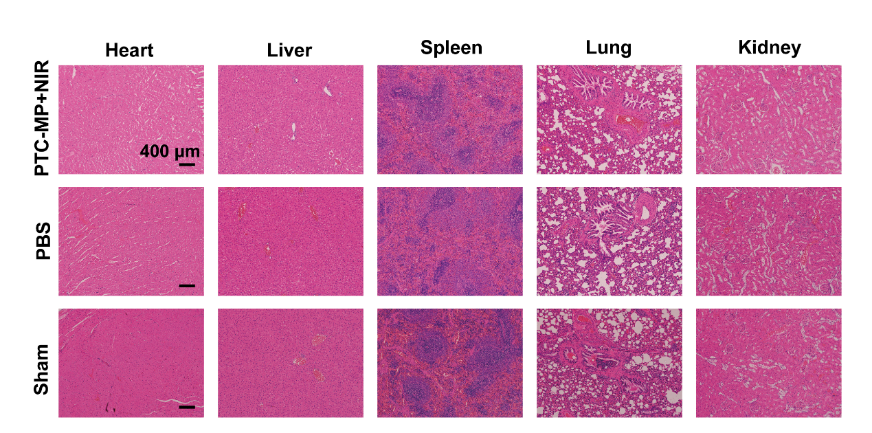
**

**Figure S37**. H&E staining of hearts, livers, spleens, lungs, and kidneys in different groups after 7 weeks.


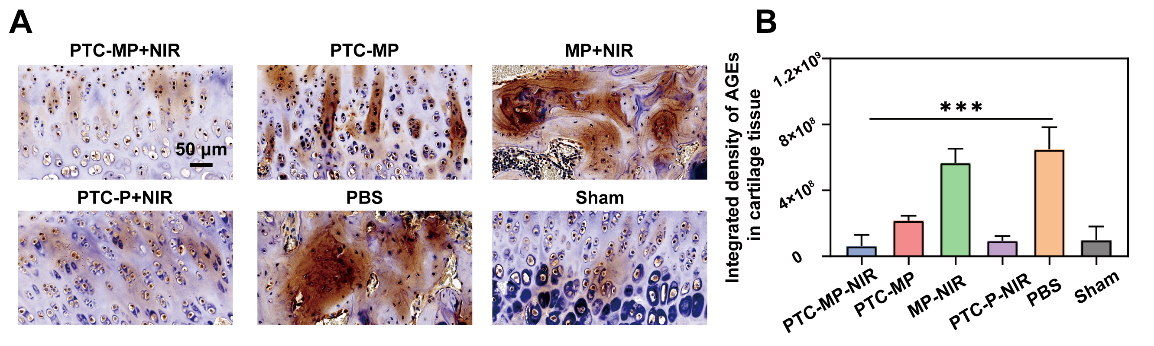


**Figure S38**. The level of AGEs expression in cartilage tissue. (A) Immunohistochemical staining of AGEs in the cartilage tissues. (B) Semi-quantitative analysis of AGEs in the cartilage tissues (n=6). Data are presented as mean values ± SD. Comparisons were performed by one-way ANOVA followed by Tukey’s multiple comparisons test. (***P < 0.001).


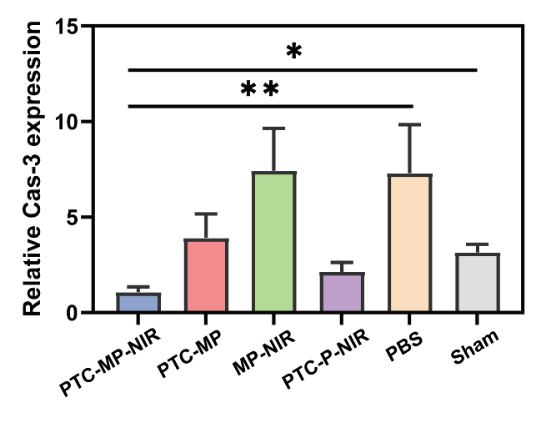


**Figure S39**. Semi-quantitative analysis of caspase-3 (Cas-3) expression in cartilage sections. (n=6). Data are presented as mean values ± SD. Comparisons were performed by one-way ANOVA followed by Tukey’s multiple comparisons test. (**P < 0.05, **P < 0.01*)


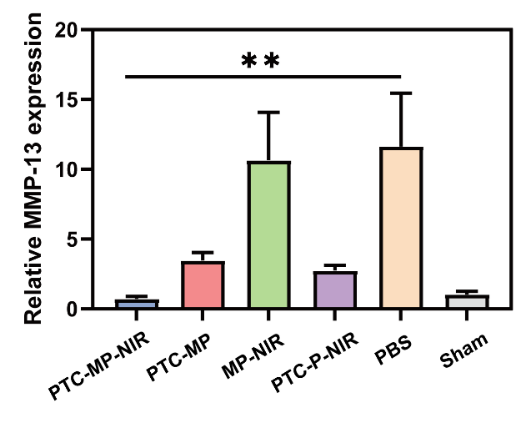


**Figure S40**. Semi-quantitative analysis of MMP-13 expression in cartilage sections. All data were shown as mean ± SD (n=6). Data are presented as mean values ± SD. Comparisons were performed by one-way ANOVA followed by Tukey’s multiple comparisons test. (***P < 0.01*)


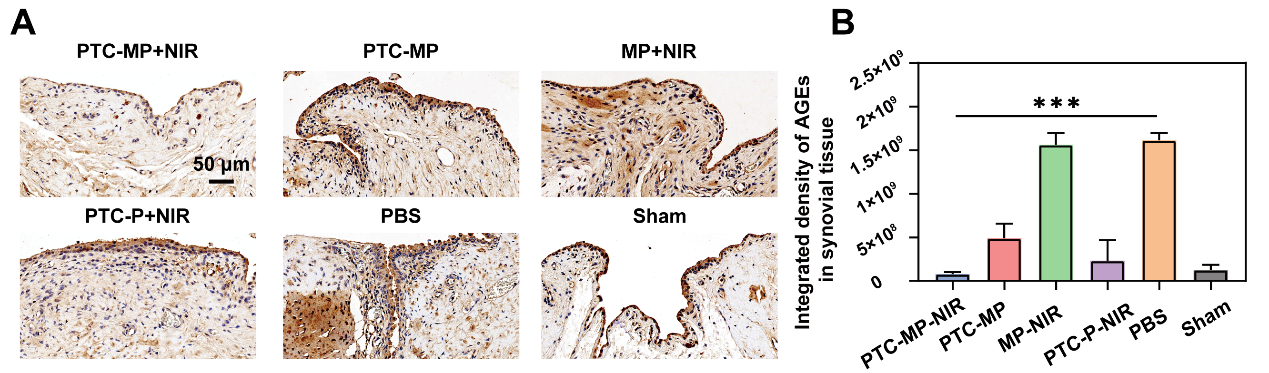


**Figure S41**. The level of AGEs expression in synovial tissue. (A) Immunohistochemical staining of AGEs in the synovial tissues. (B) Semi-quantitative analysis of AGEs in the synovial tissues (n=6). Data are presented as mean values ± SD. Comparisons were performed by one-way ANOVA followed by Tukey’s multiple comparisons test. (***P < 0.001).

**References**

[1] P. Yu, Y. Li, H. Sun, H. Zhang, H. Kang, P. Wang, Q. Xin, C. Ding, J. Xie, J. Li, *Adv. Mater.* **2023**, *35*, e2303299.

[2] S. Adisakwattana, W. Sompong, A. Meeprom, S. Ngamukote, S. Yibchok-anun, *Int. J. Mol. Sci.* **2012**, *13*, 1778.

1. *Corresponding author at: Key Laboratory of Marine Drugs, Ministry of Education, School of Medicine and Pharmacy, Ocean University of China, Qingdao 266003, China. E-mail address: [hanlu@ouc.edu.cn](mailto:hanlu@ouc.edu.cn) (L. Han); [zhaoxia@ouc.edu.cn](mailto:zhaoxia@ouc.edu.cn) (X. Zhao). [↑](#footnote-ref-1)
